# Guide to lower extremity radiologic measurements: part 2 knee

**DOI:** 10.1007/s00256-026-05187-2

**Published:** 2026-03-18

**Authors:** Allison M. Crone, Imran M. Omar, Allison M. Bronson, Lucas T. Buchler, Ryan S. Selley, Jennifer S. Weaver, Andrea S. Klauser, Mihra S. Taljanovic

**Affiliations:** 1 Department of Radiology and Imaging Sciences, Indiana University School of Medicine, 714 North Senate Ave., Suite 200, Indianapolis, IN 46202 USA; 2 Department of Radiology, Northwestern University, Feinberg School of Medicine, 676 North Saint Clair St., Suite 800, Chicago, IL 60611 USA; 3 Department of Orthopaedic Surgery, Northwestern University, Feinberg School of Medicine, 676 North Saint Clair St., Suite 1350, Chicago, IL 60611 USA; 4 Department of Radiology, University of Texas at San Antonio, Floyd Curl Drive, San Antonio, TX 78229 USA; 5 Department of Radiology, Innsbruck Medical University, Privatpraxis Bruneckerstr 2E/5, Stock, Innsbruck, Austria; 6 Departments of Radiology and Imaging Sciences and Orthopaedic Surgery, The University of Arizona, College of Medicine, 1501 North Campbell Ave, Tucson, AZ 85724 USA; 7 Department of Radiology, University of New Mexico, 1 University of New Mexico, MSC 10 5530, Albuquerque, NM 87131 USA

**Keywords:** Knee, Measurement, Radiography, CT, MRI, Patellofemoral maltracking

## Abstract

Although most imaging assessments are made qualitatively, quantitative measurements in orthopedic imaging are becoming more important in detecting subtle findings and assessing degrees of abnormality, which can help direct surgical management. In the knee, patellofemoral maltracking is a common cause of anterior pain, particularly in younger patients. Failure to recognize this pathophysiology may result in accelerated chondral loss and osteoarthritis (OA), and the imaging findings may be subtle. As a result, clinicians and radiologists have developed numerous measurements to detect and quantify patellofemoral alignment. Additional entities, such as femorotibial subluxation or angular abnormalities, may only be detected by using standardized measurements and can help detect ligamentous insufficiency or developmental malalignment, which can lead to instability and OA in the medial and lateral femorotibial compartments. Finally, the proper positioning and hardware selection for knee arthroplasty are critical to preventing early hardware failure and postsurgical pain. This review, which focuses on the knee, is the second in a three-part series discussing the appropriate imaging modalities on which to obtain specific lower extremity measurements as well as proper measurement techniques, grouped by pathology. Furthermore, the normal value or range of values according to current literature is reported, along with the significance of abnormal measurements.

## Introduction

While many diagnoses can be made through qualitative evaluation of imaging of the knee, quantitative measurements are often useful in diagnosing subtle abnormalities, grading abnormalities, guiding treatment decisions, and planning surgical interventions. For example, inaccurate measurements in patients with patellofemoral maltracking can lead to underdiagnosis or inadequate treatment, while overdiagnosis can lead to overly aggressive treatment, including unnecessary surgery. Improper measurements in planning knee arthroplasties can result in imbalanced forces on the prosthesis and premature device failure or failure of surrounding soft tissue restraints. It is important that interpreting radiologists are familiar with the correct techniques for making these measurements and understand how to interpret the values obtained. Established and standardized measurement techniques and normal values should be utilized to avoid interpretation errors and their potential implications on treatment decisions. This review article will discuss many of the commonly utilized measurements of the knee and how best to assess them on imaging.

## Imaging modalities

### Radiography

Properly positioned radiographs are a mainstay for assessing patellofemoral alignment and provide the best opportunity to obtain reproducible quantitative measurements. Therefore, it is critical that radiographs used to obtain measurements are centered over the joint being evaluated to avoid parallax from beam divergence, that the knee is not excessively internally or externally rotated especially on the lateral radiograph to allow clear depictions of the articular surfaces, and that there is strict adherence to the degree of flexion for particular projections, which is especially important to provide the appropriate degree of patellofemoral engagement. The two most commonly used projections for measurements are the Merchant and true lateral knee radiographs.

On a true lateral radiograph, the knee is positioned so that its lateral surface is close to the image detector, the knee is mildly flexed to about 30°, and the X-ray beam is centered over the femorotibial joint or the weightbearing region of the femoral condyles. The posterior margins of the femoral condyles should either be superimposed on one another or should not diverge by more than 2 mm. This allows assessment of patellar height and trochlear morphology [1].

An axial view of the knee is obtained, typically with the knee flexed between 30° and 45°, placing the patella and patellofemoral joint in profile in order to assess abnormalities of trochlear morphology, patellar tilt, and patellar subluxation. Two axial views of the knee that are particularly important for measurements are the Merchant and skyline (Laurin) radiographs. The Merchant view is performed with the patient seated with the knee flexed to 45°; the X-ray beam is directed 30° inferior to the femoral plane through the patellofemoral joint and base of the patella, providing profiles of the femoral trochlea and patella [2]. Care should be taken to avoid deeply flexing the knee during this view. In a well-positioned view, the patellar and trochlear articular surfaces should be crisply seen in profile, and the tibial tuberosity should not project over the patellofemoral joint [1].

Similarly, the skyline radiograph is an axial radiograph obtained with the knee in 20° of flexion, allowing for the position of the patella to be evaluated prior to its engagement by the femoral trochlea, when only the soft tissue stabilizers are engaged [2].

### Cross-sectional imaging

Computed tomography (CT) and magnetic resonance imaging (MRI) are useful imaging modalities to assess patellofemoral alignment and morphology, especially because these modalities are not as dependent on proper patient positioning as radiography. Imaging should include the entire knee joint from the suprapatellar region through the tibial tuberosity. Both CT and MRI can be helpful to detect joint effusions and muscle atrophy, which can contribute to patellofemoral malalignment, while MRI can better assess additional soft tissue structures that help preserve patellofemoral alignment, including the quadriceps and patellar tendons, medial and lateral patellofemoral ligaments, and medial and lateral patellar retinacula.

## Measurements (Table 1)

**Table 1:** Commonly used measurements in the radiologic assessment of the knee

Radiologic measurement	Definition	Imaging modality of choice	Normal value or normal range	Abnormal value or clinical significance
Trochlear sulcus angle	Angle formed by line extending from the deepest part of the trochlear sulcus to the anterior margins of the medial and lateral femoral condyles (Fig. 1)	Merchant radiograph of the knee	130°–145°	> 145° dysplasia
Trochlear depth	AP distance between the trochlear floor and anterior medial femoral condyle margin (Figs. 2 and 3)	Lateral knee radiograph	4–5 mm	< 4 mm dysplastic
Ventral prominence of the trochlear floor	Distance between a line tangent to the anterior femoral diaphysis and a parallel line along the anterior most margin of the trochlear floor (Fig. 5)	Lateral knee radiograph	< 3 mm	≥ 3 mm positive translation suggests trochlear dysplasia
Lateral trochlear inclination	Angle formed the posterior femoral condylar line and a line tangent to the lateral trochlear facet subchondral bone plate (Fig. 6)	Axial knee MRI through the superior margin of trochlear cartilage	≥ 11°	< 11° dysplastic trochlea
Trochlear facet asymmetry	Length of the medial trochlear facet divided by the length of the lateral trochlear facet × 100 (Fig. 7)	Axial knee MRI 3 cm above femorotibial joint	≥ 40%	< 40% dysplastic trochlea
Lateral patellofemoral angle	Angle formed by the anterior femoral condylar line and a line from the patellar median ridge along the posterior margin of the lateral facet (Fig. 8)	Merchant radiograph of the knee	≥ 8°	< 8° excessive lat. patellar tilt
Patellar inclination/tilt angle	Angle formed by the anterior femoral condylar line and a line connecting the medial and lateral edges of the patellar facets (Fig. 9)	Merchant radiograph of the knee	≤ 7°–10° on XR ≤ 10° on MRI	> 10° excessive lat. patellar tilt
Congruence angle	Angle formed by a line bisecting the sulcus angle and a line extending from the deepest part of the trochlear sulcus to the apex of the patellar median ridge (Fig. 10)	Merchant radiograph of the knee or axial cross-sectional image at mid-patellar level	≤ 16°	> 16° lateral patellar subluxation
Lateral patellar displacement	Transverse distance between the medial border of the patella and apex of the medial femoral condyle (Fig. 11)	Merchant radiograph of the knee or axial cross-sectional images at medial border of the patella and medial margin of femoral trochlea	< 5 mm	≥ 5 mm lateral patellar subluxation
Insall–Salvati index	Ratio of the patellar tendon length (inferior patellar margin to the tibial tuberosity) divided by the patellar length (Fig. 12)	Lateral knee radiograph; can use sagittal MR image of knee through the patella and patellar tendon	0.8–1.3 on XR	**·** > 1.3 patella alta **·** < 0.8 patella baja
Modified Insall–Salvati index	Ratio of the distance from the inferior margin of the patellar articular surface to the tibial tuberosity and the patellar articular surface length (Fig. 13)	Lateral knee radiograph; can use sagittal MR image of the knee through the patella and patellar tendon	1.2–2.0	> 2.0 patella alta
Canton–Deschamps index	Ratio of the distance from the inferior margin of the patellar articular surface to the anterior margin of the tibial articular surface to the patellar articular surface length (Fig. 14)	Lateral knee radiograph; can use sagittal MR image of knee through the patella and patellar tendon	1.0	> 1.3 patella alta < 0.6 patella baja
Blackburne–Peel ratio	Ratio of the patellar height (distance from a horizontal line along the tibial plateau and the inferior patellar margin) divided by the length of the patellar articular surface (Fig. 15)	Lateral knee radiograph; can use sagittal MR image of the knee through the patella and patellar tendon	0.8	> 1.0 patella alta
Patellotrochlear index	Ratio of the craniocaudal length of the trochlear cartilage overlapped by patellar cartilage to the patellar articular surface length (Fig. 16)	Sagittal MRI at the level of the maximal patellar cartilage thickness	~0.32 average	**·** < 0.12–0.28 patella alta **·** > 0.50–0.80 patella baja
Anterior tibial subluxation	Distance between the posterior lateral femoral condylar subchondral bone plate and the posterior lateral tibial plateau on a sagittal MR image through the mid-lateral compartment (Fig. 17)	Sagittal image of knee at mid-lateral compartment level	≤ 7 mm	> 7 mm anterior tibial subluxation
Tibial tuberosity-trochlear groove distance	Distance between the tibial tuberosity and trochlear groove, measured parallel to the posterior trochlear line (Fig. 18)	Axial CT or MRI through the trochlear sulcus and tibial tuberosity	< 15 mm	**·**15–20 mm borderline **·** > 20 mm suggests lateral displacement of tibial tuberosity

### Trochlear dysplasia

The patellofemoral joint relies on proper osseous and soft tissue morphology for biomechanical stability during knee flexion and extension. In the setting of trochlear dysplasia, in which the trochlear sulcus is abnormally shallow, there is deficient engagement of the patella by the trochlear sulcus during knee flexion. As a result, there is decreased patellar stability within the trochlear groove, which may allow the patella to subluxate laterally under the influence of strong lateral stabilizers, including the vastus lateralis muscle. This predisposes patients to recurrent lateral patellar subluxation/dislocation and to the development of patellofemoral chondromalacia and osteoarthritis (OA) [1]. The overall prevalence of trochlear dysplasia in skeletally mature patients has been reported at 4.5% using CT and 6% using ultrasound [3, 4]. However, its prevalence has been reported as 85% on CT in patients with patellar instability requiring surgical correction [5].

Dejour et al. classified dysplastic trochleae into four categories (A–D) based on morphology, which are assessed on a combination of true lateral knee radiographs and axial knee CT or MRI. Dejour type A trochleae represent low-grade trochlear dysplasia and are seen in about 15–20% of patients. These trochleae are concave but shallow and are diagnosed on lateral knee radiographs when there is a *crossing sign* between the anterior margin of the femoral condyles and the deepest part of the trochlear sulcus. Dejour type B trochleae are the most common type, representing between 28 and 42% of patients. This morphology is flat and reflects high-grade trochlear dysplasia; it is diagnosed on lateral radiographs on which there is a supratrochlear spur in addition to the crossing sign. Type C and D morphologies are also high grade and reflect convex trochleae. Axial imaging of the knee is generally needed to discriminate between these morphologies. Type C trochleae represent 13–16% of patients and are associated with medial trochlear hypoplasia that is best detected on cross-sectional imaging. On lateral radiographs, this morphology leads to a crossing sign as well as a double density inferior to the point of crossover, which indicates the medial trochlear facet is posterior to the lateral facet. Finally, type D trochleae are seen in 4–17% of patients and have a medial trochlear cliff. This morphology is associated with a supracondylar spur, a crossing sign, and an inferior trochlear double density [6]. In practice, it may be best to simply discriminate between Dejour low- and high-grade trochlear dysplasia, since there is only slight interobserver reliability in distinguishing between the three types of high-grade trochlear dysplasia [7].

A newer classification system, the Oswestry–Bristol Classification system, which has a four-part classification of trochlear morphology, has shown better interobserver agreement and may be more practical for clinical assessment [8]. This scheme divides trochlear morphology into normal, mild trochlear dysplasia when there is mild concavity, moderate trochlear dysplasia when the morphology is flat, and severe when the morphology is convex [9].

#### Trochlear sulcus angle

**Fig. 1:**
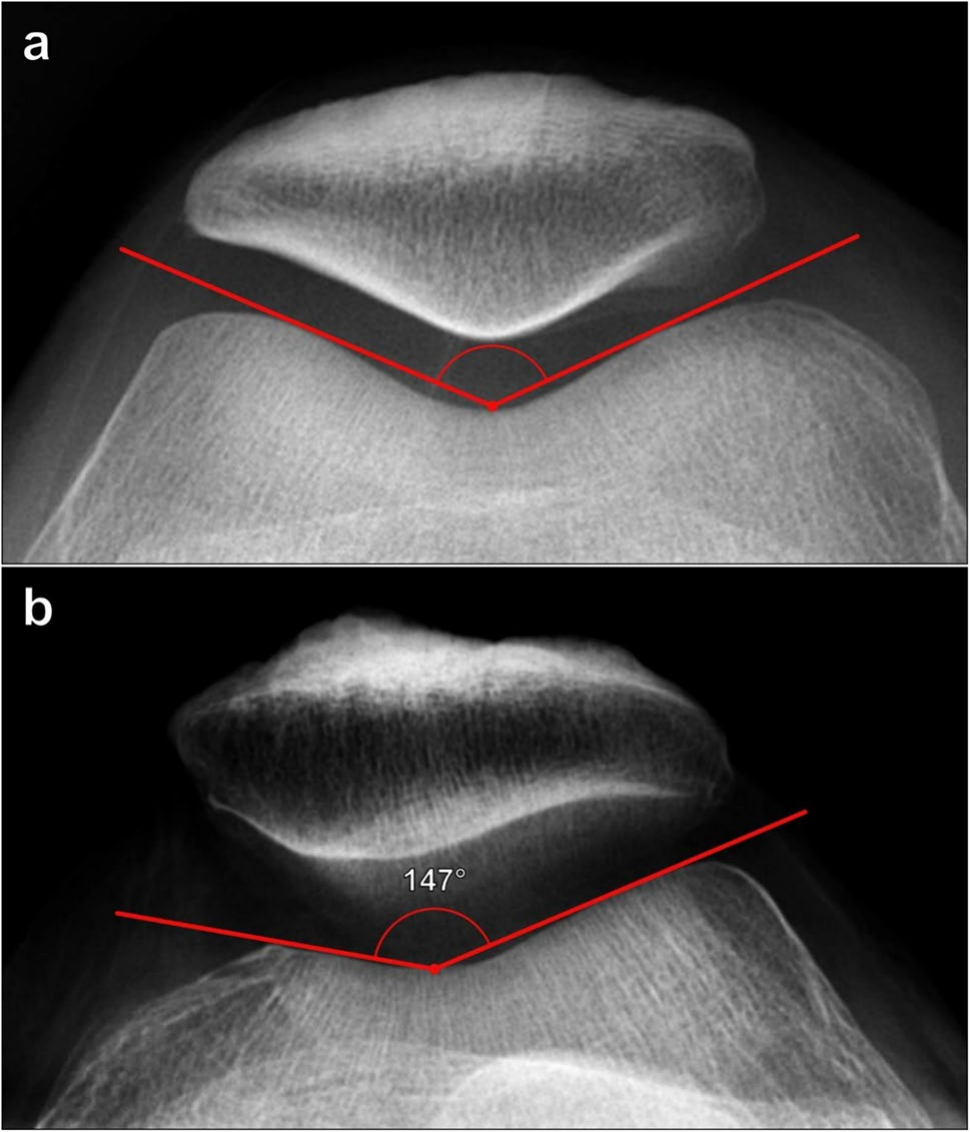
Measurement of trochlear sulcus angle on Merchant radiographs. **a** The angle is formed from the deepest point of the trochlear sulcus with arms drawn along the anterior margins of the femoral condyles. The normal range is 130°–145° with a trochlear sulcus angle > 145° suggesting trochlear dysplasia. In this normal example, the angle measures 135°. **b** In a 37-year-old female patient with anterior knee pain, the sulcus angle measures 147°, which is increased and indicates trochlear dysplasia

The trochlear sulcus angle represents the steepness of the trochlear groove, with wider angles indicating a flatter groove. The angle is measured on a Merchant radiograph and is formed by lines drawn from the deepest portion of the trochlear sulcus to the most anterior margins of the medial and lateral femoral condyles (Fig. 1). The normal range of values is 130° to 145° with a sulcus angle > 145° suggesting trochlear dysplasia [5]. This measurement can also be reliably assessed on both CT and MRI utilizing the same measurement technique [10]. When performed on cross-sectional imaging, it should be done about 3 cm superior to the femorotibial joint, where the trochlear groove should be best defined. On CT, the position should be confirmed either on initial localizer planar images or on sagittal or coronal reformatted images in order to maintain proper measurement standardization.

#### Trochlear depth

The depth of the trochlear sulcus is an important determinant of trochlear dysplasia. Trochlear depth can be measured on true lateral radiographs of the knee, on which the trochlear floor is visualized as a radiodense arc projecting posterior to the anterior femoral condyles. The linear measurement between this arc and the anterior margin of the lateral femoral condyle is indicative of the trochlear depth.

**Fig. 2:**
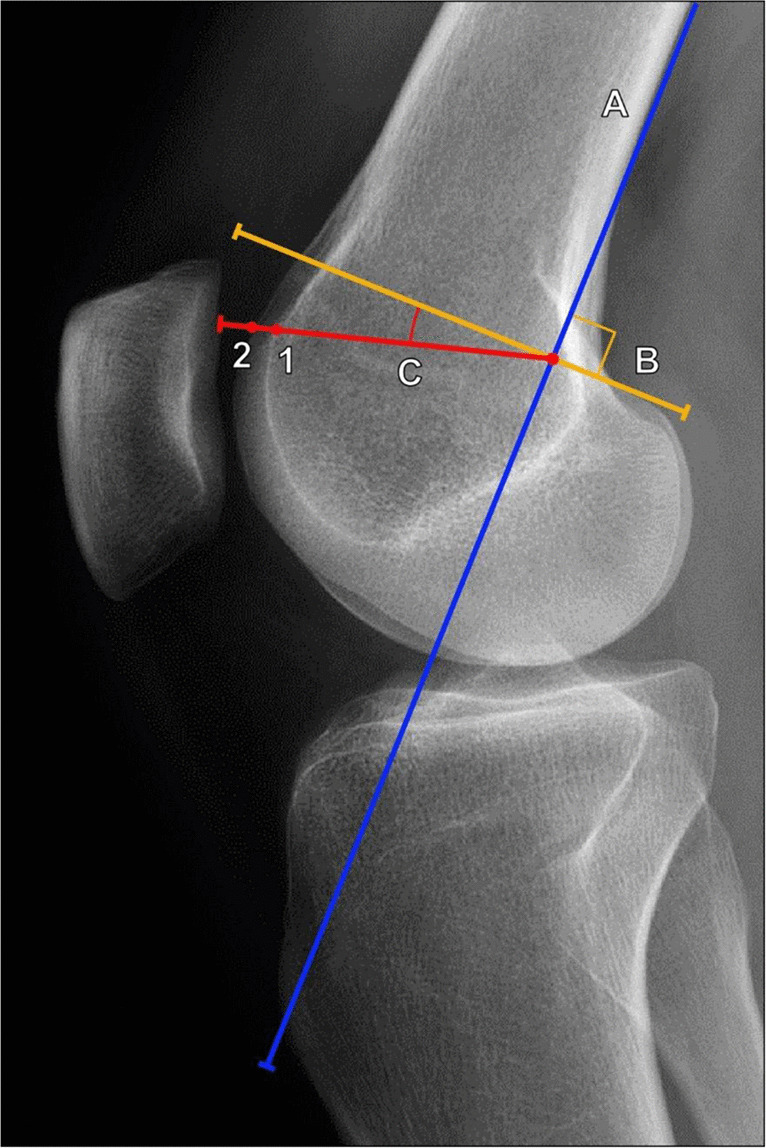
Measurement of trochlear depth on lateral radiograph of the knee utilizing Dejour’s method. A line (A, blue line) tangent to the posterior femoral cortex is drawn, and a line (B, yellow line) is drawn perpendicular to this at the most superior margin of the posterior femoral condyle. A line 15° inferior to line B is drawn (C, red line), which extends through the trochlear line (1) and the most anterior margin of the lateral femoral condyle (2). The distance between 1 and 2 is the calculated trochlear depth. A trochlear depth < 4 mm indicated trochlear dysplasia

Dejour et al. described a process to standardize the measurement of the trochlear depth on lateral radiographs by drawing a line along the posterior femoral diaphyseal cortex and a second, perpendicular line to the first at the level of the most proximal portion of the posterior femoral condyle. Subsequently, a third line is extended 15° anteroinferior from the perpendicular line, and the measurement from the anterior lateral femoral condyle and the trochlear floor is made along this line (Fig. 2). Using this method, a trochlear depth of < 4 mm is considered abnormal [5].

**Fig. 3:**
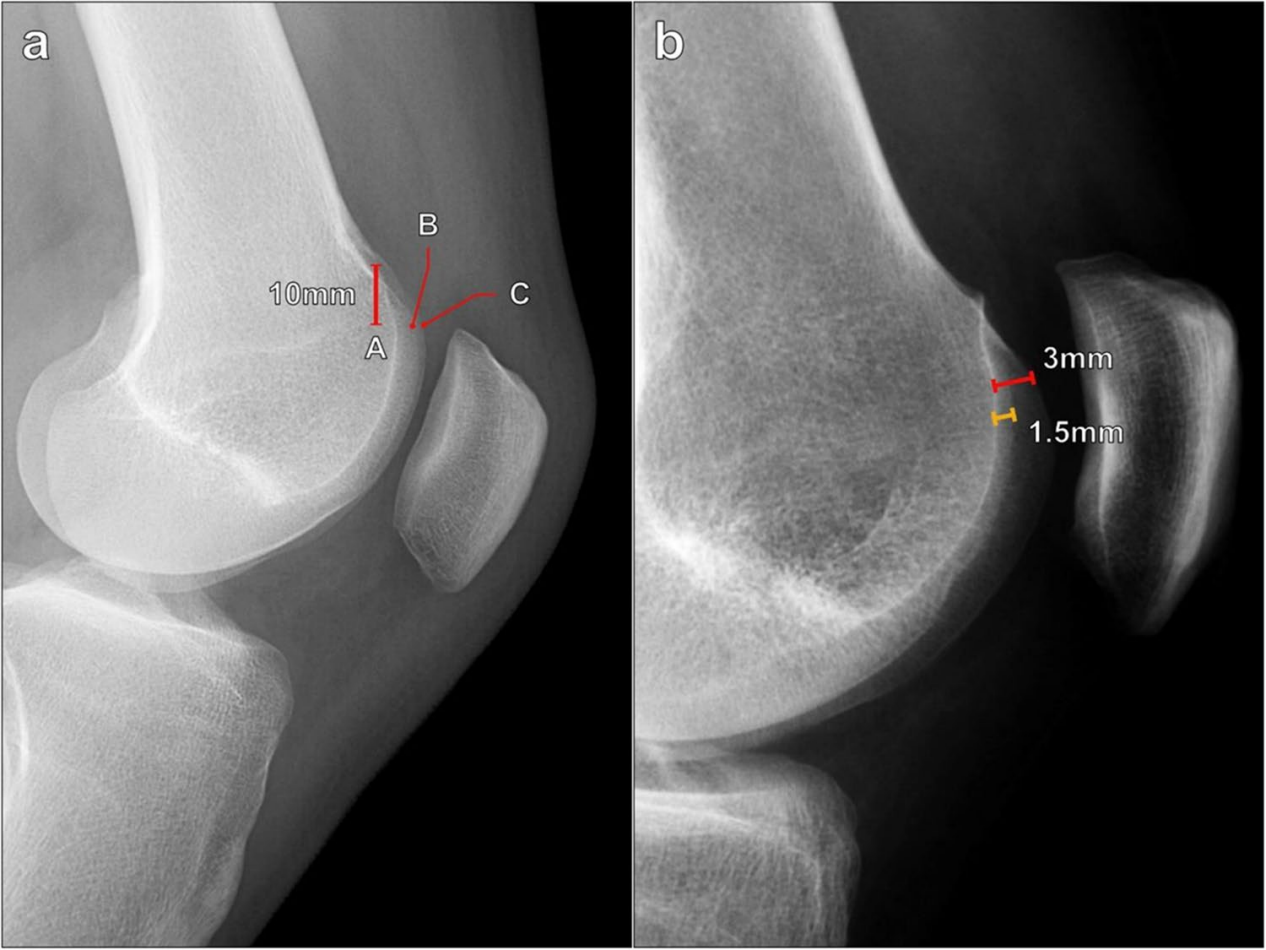
Measurement of trochlear depth on lateral knee radiograph utilizing Malghem’s method. **a** The trochlear depth measurement is made at a level 10 mm inferior to the superior trochlear sulcus, between the trochlear floor (A) and the anterior margins of the medial femoral condyle (B) and the lateral femoral condyle (C), and the average of these is calculated. Values < 5 mm indicate trochlear dysplasia. In this example, the trochlear depth measures 5 mm, which is normal. **b** In a 37-year-old female patient with anterior knee pain, the trochlear depth measures 3 mm using the anterior margin of the lateral femoral condyle (red line), which already meets the criteria for trochlear dysplasia. However, using Malghem’s method, the distance to the anterior margin of the medial femoral condyle measures 1.5 mm (yellow line), and the average of the two is 2.3 mm

Malghem et al. described a similar method for measuring trochlear depth on a lateral radiograph, which is made 10 mm below the superior margin of the trochlear sulcus between the trochlear floor and the anterior margin of the lateral femoral condyle and the anterior margin of the medial femoral condyle (Fig. 3). The average of these two measurements is then taken. In practice, on a well-positioned lateral radiograph, the anterior margins of the femoral condyles typically overlap in such a way that the two values are nearly equivalent; however, this method can help to correct for slight variations in internal or external rotation of the knee. Malghem et al. considered measurements < 5 mm indicative of a shallow trochlear groove [11].

**Fig. 4:**
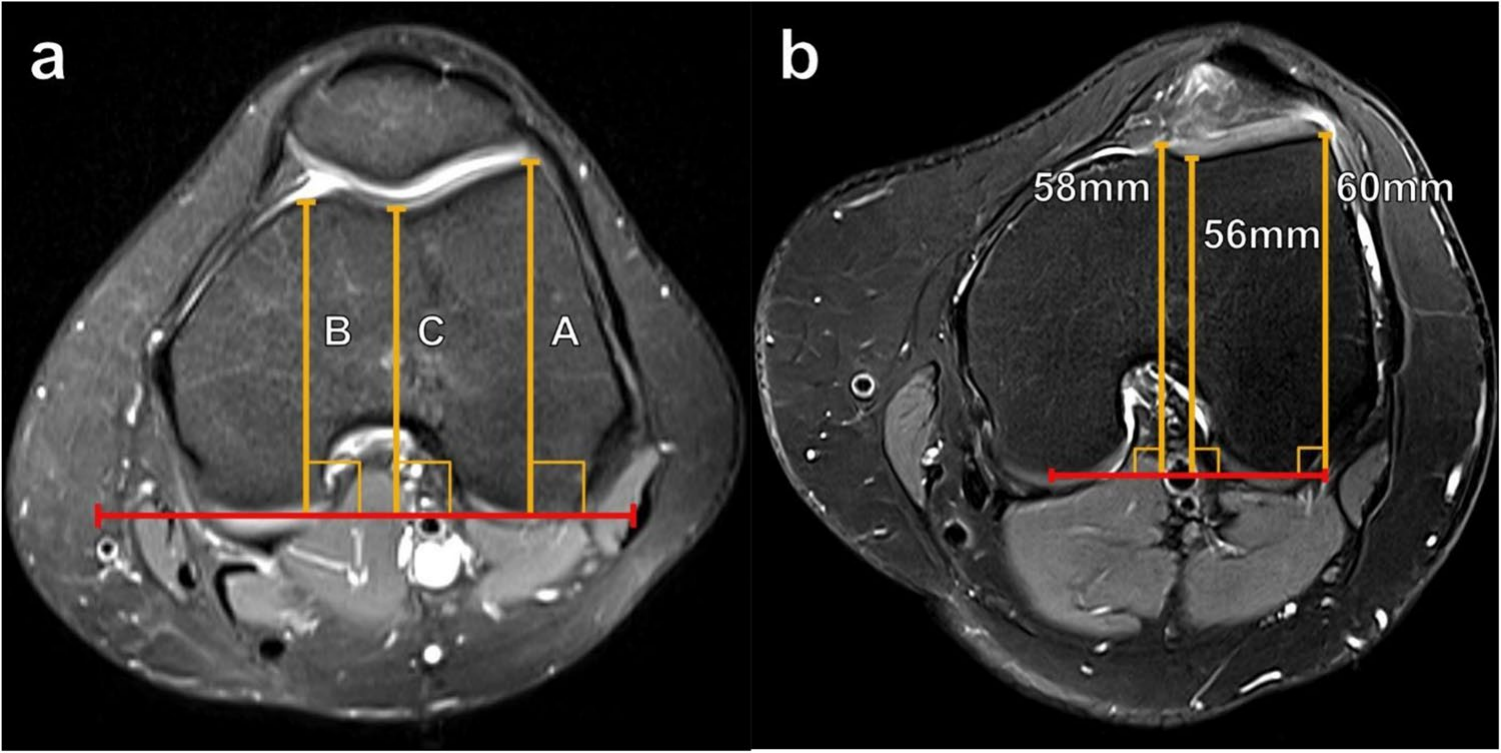
Measurement of trochlear depth on axial T2 fat-suppressed MR images of the knee. **a** Lines anteriorly from the posterior condylar line (red line) to the anterior margins of the lateral femoral condyle (A), the medial femoral condyle (B), and the trochlear floor (C) at a level 3 cm superior to the femorotibial joint. The trochlear depth is calculated as [(A + B)/2] − C. Values < 3 mm indicate trochlear dysplasia. **b** In a 28-year-old female patient with anterior knee pain, the trochlear depth measured just less than 3 mm, indicating trochlear dysplasia

MRI techniques for measuring trochlear depth, such as the one described by Pfirrmann et al., have demonstrated very good reliability [10]. As depicted in Fig. 4, this technique utilizes an axial image of the knee approximately 3 cm proximal to the femorotibial joint line. First, a line is drawn connecting the posterior femoral condyles. The maximum anteroposterior (AP) distances are then obtained from the posterior condylar line to the anterior lateral femoral condyle (A) and to the anterior medial femoral condyle (B), and the average of these distances is calculated. Then, the minimal AP distance from the posterior femoral condylar line to the deepest part of the trochlear floor (C) is obtained and subtracted from the average condylar AP distance. Thus, the trochlear depth is calculated using the formula [(A + B)/2] − C. A calculated trochlear depth < 3 mm using this method has been found to be sensitive and specific for trochlear dysplasia [12]. Appropriate slice selection is critical to standardize the measurement, improve interobserver reliability, and avoid underestimation. Additionally, it is important to note that MRI assessment of trochlear depth should be performed with respect to the trochlear hyaline cartilage rather than the subchondral bone plate to avoid underdiagnosing trochlear dysplasia.

#### Ventral prominence of the trochlear floor (trochlear bump measurement)

**Fig. 5:**
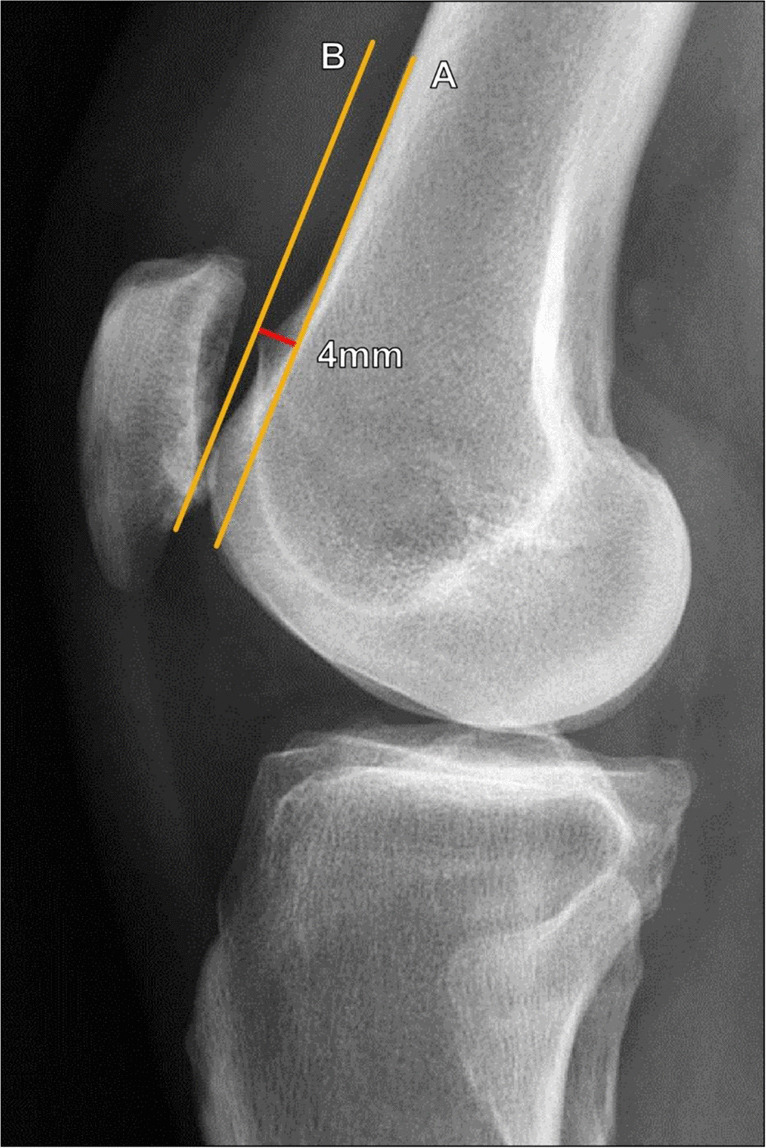
Measurement of ventral prominence of the trochlear floor on lateral radiograph of the knee. A line (A) is drawn tangent to the anterior distal femoral diaphysis, and a second parallel line (B) is drawn at the level of the anteriormost margin of the trochlear floor. The distance between these two lines is the ventral prominence of the trochlear floor. A measurement ≥ 3 mm indicates trochlear dysplasia. Incidentally, it should be noted that this radiographic value is separate from the MRI-based ventral prominence, which should be ≤ 8 mm

This measurement assesses the most anterior aspect of the trochlear floor relative to the anterior distal femoral diaphyseal cortex. In patients with trochlear dysplasia, there is often abnormal ventral prominence of the trochlear floor, which forms a “trochlear bump,” as described by Dejour et al. [5]. To quantitatively assess this finding on a true lateral knee radiograph, a tangent line along the distal 10 cm of the anterior femoral cortex extending distal to the articular surface is drawn, and a second, parallel line is created along the anterior-most margin of trochlear floor (Fig. 5). The trochlear floor can be in line with the anterior femoral cortex line (no translation), anterior to this line (positive translation), or posterior to this line (negative translation). In most people, there is either no translation or slightly negative translation. A positive translation of ≥ 3 mm on radiography is suggestive of trochlear dysplasia. [5]. This can only be measured on a true lateral knee radiograph since any rotation can obscure this finding and make the measurements inaccurate.

This measurement can also be made on cross-sectional imaging at the mid-trochlear sagittal plane. Pfirrmann et al. described a technique that measured the distance between a line along the ventral femoral diaphyseal cortex and the most ventral cartilaginous surface of the trochlear floor. Values ≥ 8 mm on MRI were found to be suggestive of trochlear dysplasia [12].

#### Lateral trochlear inclination

**Fig. 6:**
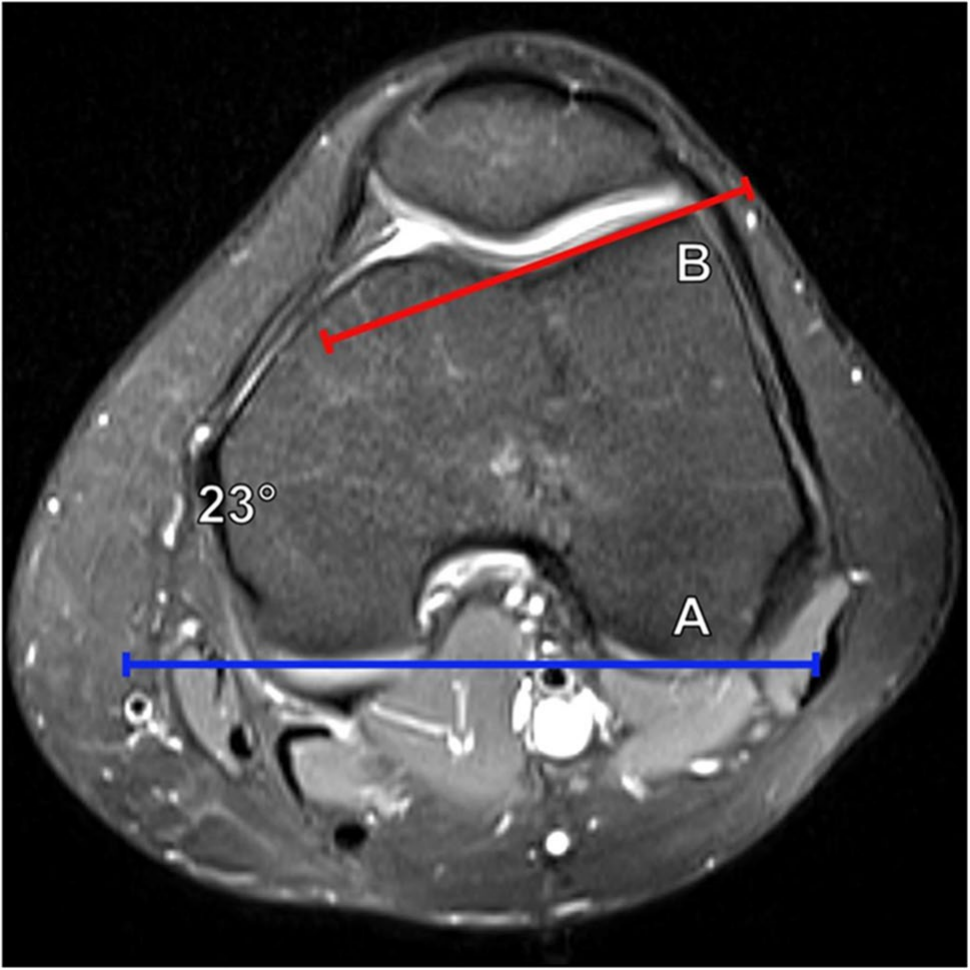
Measurement of lateral trochlear inclination on axial T2 fat-suppressed MR image of the knee. The angle is formed between the posterior condylar line (A, blue line) and a line tangent to the lateral trochlear facet (B, red line). A measurement of < 11° indicates trochlear dysplasia

Initially described as a measurement on axial radiographs [13], the lateral trochlear inclination is now primarily measured on cross-sectional imaging. The MRI measurement of the lateral trochlear inclination was initially described by Carrillon et al. The measurement is made on axial imaging of the knee, at the most superior level where trochlear cartilage is visualized. The lateral trochlear inclination angle is formed between a line along the subchondral bone of the posterior femoral condyles and a line tangential to the subchondral bone of the lateral trochlear facet (Fig. 6). An angle < 11° is considered abnormal and suggests trochlear dysplasia [14].

#### Trochlear facet asymmetry

**Fig. 7:**
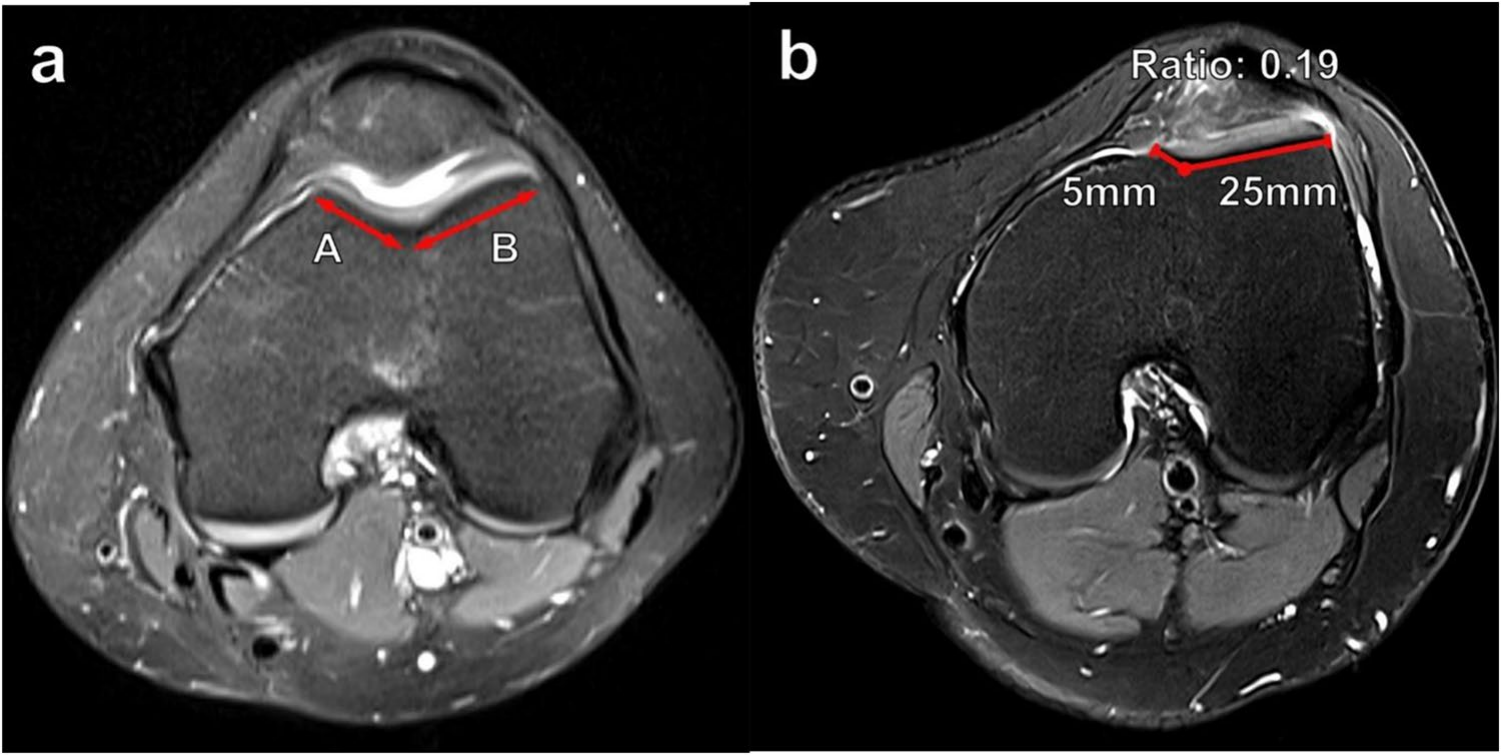
Measurement of trochlear facet asymmetry on axial T2 fat-suppressed MR images of the knee. **a** At a level 3 cm proximal to the femorotibial joint, the length of the medial facet (A) and lateral facet (B) is measured. The medial facet length is divided by the lateral facet length and multiplied by 100% [(A/B) × 100]. A facet ratio of < 40% suggests trochlear dysplasia. In this example, the trochlear facet asymmetry ratio measures 60%, which is normal. **b** On a 28-year-old female patient with anterior knee pain, the ratio measures only 19%, suggesting trochlear dysplasia. Additionally, there is superolateral Hoffa’s fat pad edema, related to lateral patellar maltracking and impingement

In the setting of trochlear dysplasia, there may be hypoplasia of the medial trochlear facet relative to a more prominent lateral trochlear facet. Pfirrmann et al. described a technique to measure facet asymmetry on an axial MR image 3 cm cranial to the femorotibial joint line, although CT can also be used. The length of the medial facet (a) and lateral facet (b) is measured. The medial facet length is then divided by the lateral facet length and multiplied by 100% to give a percentage [(a/b) × 100%] (Fig. 7). Values < 40% were found to be highly sensitive and specific for trochlear dysplasia [12]. Greater degrees of trochlear facet asymmetry generally correspond to Dejour type C and D morphology, in which the medial trochlear facet is markedly hypoplastic.

## Patellofemoral alignment

Patellofemoral malalignment in the transverse plane can lead to increased contact forces between the patella and the femoral trochlea during knee extension and flexion, which can result in accelerated articular cartilage loss and OA. In most cases, there are greater laterally directed forces on the patella, leading to lateral patellar maltracking, lateral patellar facet overload, and lateral-predominant chondral loss. Patellofemoral maltracking is generally multifactorial, often occurring through a combination of osteoarticular factors, such as trochlear dysplasia, and soft tissue conditions, such as a strong, laterally directed force by the vastus lateralis muscle, or medial patellar retinacular insufficiency. Associated imaging findings include lateral patellar tilt, lateral patellar subluxation, or a combination of both.

### Patellar tilt

Patellar tilt represents a laterally directed rotational torque on the patella in the axial plane that results from lateral patellar retinacular stiffness and/or medial patellar ligamentous insufficiency. Abnormal lateral patellar tilt without subluxation is a defining feature of *excessive lateral pressure syndrome*, which is a common cause of anterior knee pain [15]. Patellar tilt is commonly measured by one of two angles, the lateral patellofemoral angle or the lateral patellar inclination angle, and it is best quantitatively assessed on either axial patellofemoral radiographs or axial MRI.

#### Lateral patellofemoral angle (of Laurin)

**Fig. 8:**
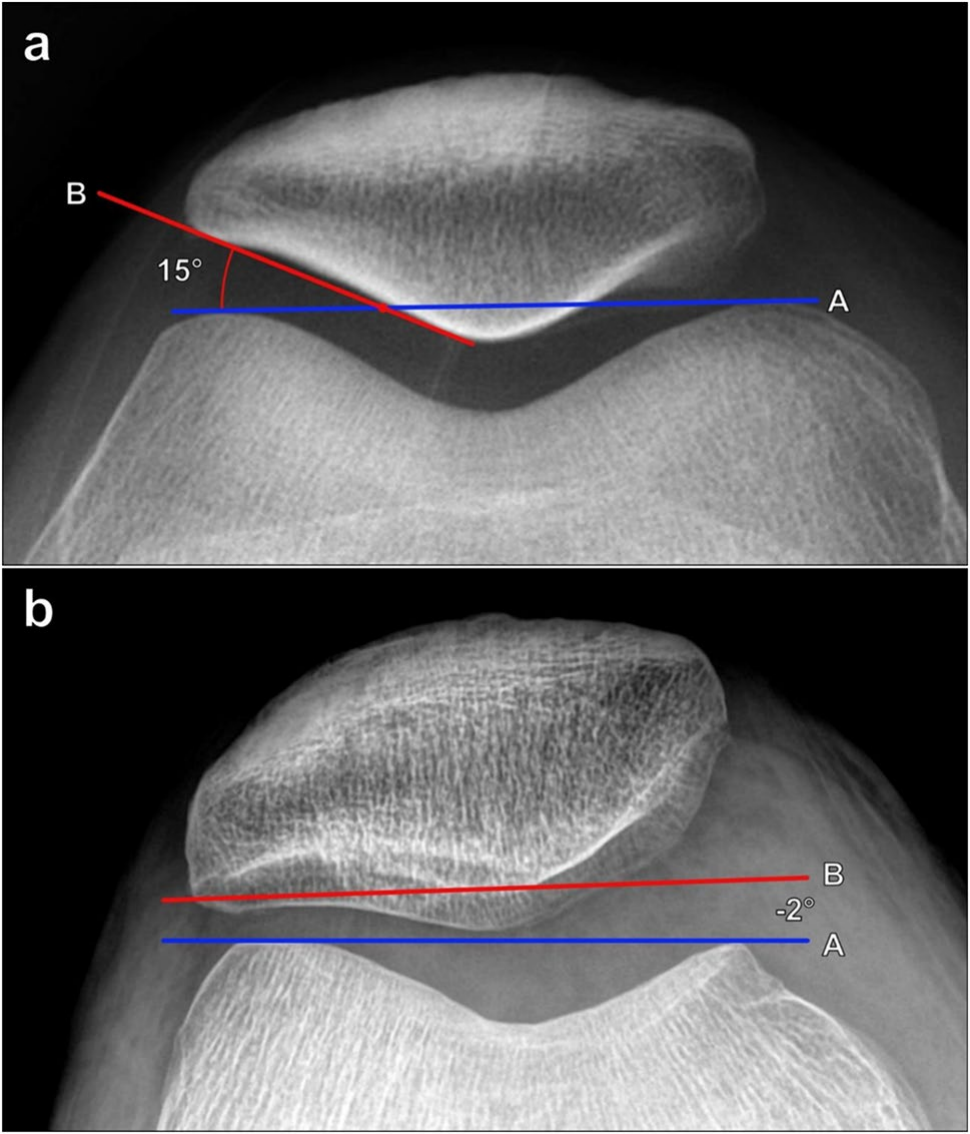
Measurement of lateral patellofemoral angle on Merchant radiograph of the knee. **a** The angle is measured between a line (A, blue line) drawn connecting the anterior margins of the femoral condyles and a line (B, red line) extending from the patellar median ridge along the posterior margin of the lateral facet. The angle should open laterally, and values < 8° or medially opening angles indicate abnormal lateral patellar tilt. **b** In a 25-year-old female patient with anterior knee pain, the angle measures −2°, indicating a medially opening angle and lateral patellar tilting

This angle is formed from two lines on axial knee radiographs (Fig. 8). Initially, it was described on the skyline view but is more commonly currently performed on a Merchant view. The first line connects the anterior margins of the femoral condyles, while the second line extends from the patellar median ridge along the lateral patellar facet [16]. The angle should open laterally (indicated by a value > 0°) and typically measures ≥ 8° [17]. A measurement of < 8°, including negative values, which indicate the angle opens medially, is considered abnormal [15, 16]. The lateral patellofemoral angle can also be measured as described above on cross-sectional imaging at the level of the mid patella. Grelsamer et al. and Wittstein et al. described measurements of the lateral patellofemoral angle in which the femoral condylar line is drawn tangent to the posterior femoral condyles rather than the anterior femoral condyles [17, 18], which are more similar to the physical examination.

#### Patellar inclination angle/patellar tilt angle

**Fig. 9:**
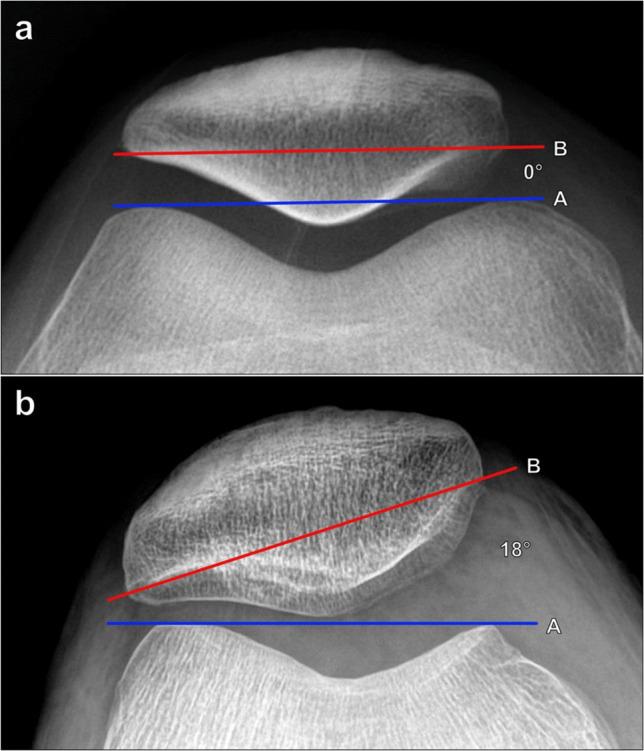
Measurement of the patellar inclination/patellar tilt angle on Merchant radiograph of the knee. **a** The angle is formed between a line (A, blue line) connecting the anterior margins of the femoral condyles and a line (B, red line) between the medial margin of the medial patellar facet and the lateral margin of the lateral patellar facet. A patellar tilt angle of > 7°–10° on radiography is considered abnormal, while an angle of > 10° on CT or MRI is considered abnormal. **b** In the same patient for Fig. 8a, the patellar tilt angle measures 18°, indicating excessive lateral patellar tilting

The second measurement used to determine excessive lateral patellar tilt is the patellar inclination/patellar tilt angle, which is measured on a Merchant knee radiograph (Fig. 9). The angle is formed by one line connecting the anterior margins of the femoral condyles and a second line connecting the medial edge of the medial patellar facet and the lateral edge of the lateral patellar facet. More greatly positive angles indicate progressive medial opening of the angle. The reported normal cutoff value has been variable. The vast majority of normal knees have a patellar tilt angle of < 15°, and initially, a patellar tilt angle of > 20° on radiography was considered abnormal [5]. However, more recently, values > 7°–10° have been utilized as cutoffs to detect excessive lateral patellar tilt.

The patellar inclination/patellar tilt angle can also be estimated on cross-sectional imaging by using a line connecting the posterior femoral condylar margins and a line connecting the medial and lateral patellar borders at the mid patella where the patella is widest, which helps to improve measurement standardization [18]. Grelsamer et al. described the MRI patellar inclination/patellar tilt angle, with an upper limit of normal of 10°; several asymptomatic patients demonstrated angles of around 10°, so utilizing this cutoff captures essentially all cases of abnormal patellar tilt but would also include patients for whom the measurement does not correlate to clinically significant findings [18]. Thus, utilizing a higher cutoff for the patellar tilt angle on cross-sectional imaging may help to better distinguish normal knees from those with clinically relevant abnormal patellar tilt.

### Patellar subluxation

Patellar subluxation reflects a linear translation of the patella with respect to the femoral trochlea in the axial plane rather than rotational malalignment. It is best assessed on axial radiographs with the knee flexed between 30° and 45°. Similar to patellar tilt, this commonly occurs laterally due to disproportionate tightness of static lateral stabilizers and greater force by dynamic lateral stabilizers, such as the vastus lateralis muscle. The relative position of the patella compared with the femoral trochlea can be assessed by either angular measurements, such as the congruence angle, or linear measurements, like lateral patellar displacement distance, and angular and linear measurements offer complementary information.

#### Congruence angle

**Fig. 10:**
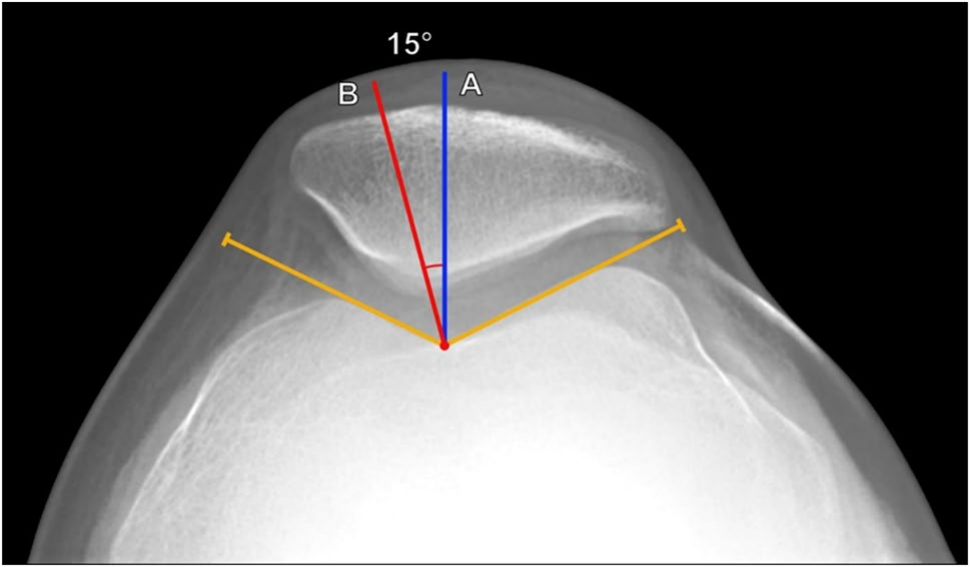
Measurement of the congruence angle on Merchant radiograph of the knee. The angle is formed between a line (A) bisecting the trochlear sulcus angle and a line (B) through the crest of the patellar median ridge

The congruence angle, initially described by Merchant et al., evaluates the relationship between the patellar median ridge and the deepest part of the trochlear groove. It is measured on an axial view of the knee, either a Merchant radiograph or on cross-sectional imaging at the mid-patellar level (Fig. 10). To measure the congruence angle, initially, the trochlear sulcus angle is measured as previously described. A line bisecting the trochlear sulcus angle is drawn, and the congruence angle is formed between this line and a second line extending anteriorly from the deepest part of the trochlear sulcus (the apex of the sulcus angle) through the crest of the patellar median ridge. If the crest of the patellar median ridge is lateral to the trochlear sulcus, the angle is positive, and if the patellar median ridge is medial to the trochlear sulcus, the angle is negative. The congruence angle is considered abnormal, indicating lateral subluxation of the patella, if it measures > 16° [19].

#### Lateral patellar displacement

**Fig. 11:**
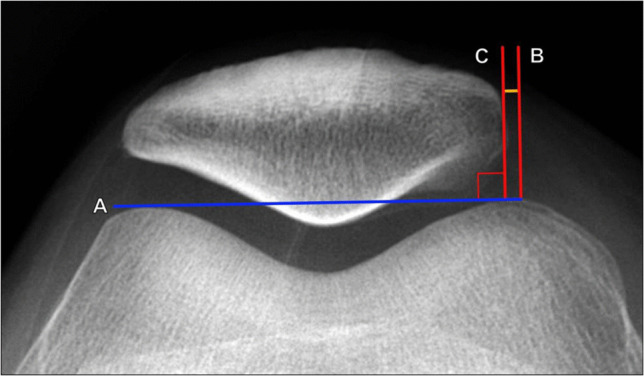
Measurement of lateral patellar displacement on Merchant radiograph of the knee. Lines are drawn perpendicular to the anterior condylar line (A, blue line) at the apex of the medial femoral condyle (B) and along the medial margin of the patella (C). The distance between these lines is the lateral patellar displacement. The most commonly reported normal value is < 5 mm

Lateral patellar displacement measures the linear distance in the axial plane between the medial margins of the patella and medial trochlear facet (Fig. 11). On the Merchant knee radiograph, a line connecting the anterior margins of the femoral condyles should be drawn. Two lines are then drawn perpendicular to the anterior condylar line: one line along the medial border of the patella and the other through the anterior margin of the medial femoral condyle [16].

This measurement can also be assessed on cross-sectional imaging. However, it requires the creation of reference lines on multiple axial images. The initial transverse reference line is taken by forming a line connecting the posterior margins of the femoral condyles. Then, as previously described, two lines perpendicular to the posterior femoral condylar line are drawn: a line intersecting the anterior margin of the medial femoral condyle and a line extending along the medial patellar margin. The patellar displacement is the distance between the two perpendicular lines. The published normal values range from ≤ 2 to < 7 mm, and many authors cite a normal cutoff of < 5 mm. CT and MRI offer better reproducibility due to standardized slice orientation; however, there may be slight differences in measured values between axial radiographs, which are performed with the knee in mild flexion, and cross-sectional imaging, which is commonly performed with the knee extended.

### Patellar height

The patellar height represents the distance between the patella and the tibial tuberosity. The patella serves as a fulcrum for the extensor mechanism and can affect the force generated by the quadriceps musculature during knee extension. There is an optimal patellar height that allows the patella to engage the femoral trochlea throughout its range of motion and to generate maximal power without resulting in pathology, including patellofemoral OA. *Patella alta* occurs when the patellar height is increased, which can lead to patellofemoral instability and maltracking, while *patella baja* occurs when the patellar tendon is shortened, which can lead to decreased force generation during extension and decreased range of motion. There are several commonly used methods to determine patellar height, which are subsequently described. In practice, these methods are largely equivalent and may be more common based on regional preferences. These indices are assessed on true lateral knee radiographs with the knee in 30° of flexion to allow consistency and reproducibility, and they are not interchangeable. Additionally, sagittal MRI through the intercondylar region and patellar tendon can be used to assess patellar height and may provide results similar to radiography. However, because the knee is usually imaged in terminal extension on MRI, which can result in patellar tendon laxity, caution should be used to ensure the patellar tendon is taut before measuring these indices [20].

#### Insall–Salvati index

**Fig. 12:**
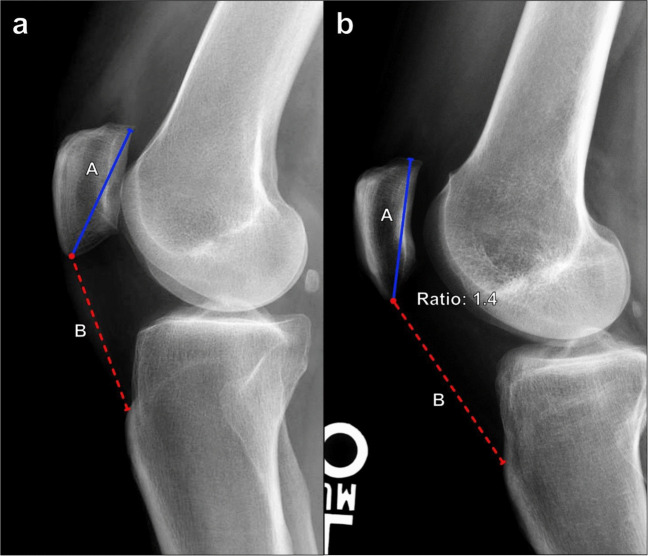
Measurement of the Insall–Salvati index on lateral knee radiograph. **a** The ratio is calculated as the maximal patellar tendon length (B, red line) divided by the maximal patellar length (A, blue line). The range of normal values is 0.8–1.3. A measured ratio of > 1.3 reflects patella alta, while a ratio < 0.8 indicates patella baja. **b** Patella alta in a 37-year-old female with anterior knee pain. The Insall–Salvati index measures 1.4, which is mildly increased

This is the most commonly used method for measuring patellar height. Because it does not depend on the degree of knee flexion, it is thought to measure the relative length of the patellar tendon the most accurately [21]. Measured on lateral knee radiographs, the Insall–Salvati ratio compares the length of the patellar tendon along its deep border from the patellar attachment to the tendon insertion on the tibial tuberosity and the maximal patellar length (Fig. 12). The patellar tendon length is divided by the patellar length, and the normal range of values is 0.8–1.3. Calculated values > 1.3 indicate patella alta, while those < 0.8 indicate patella baja [22]. On MRI, different thresholds are used, with normal ranging from 0.74 to 1.5 [23].

It should be noted that this method of assessing patellar height is very sensitive to patellar and tibial tuberosity morphology. The lower pole of the patella can often be developmentally elongated even though the articular surface remains relatively unchanged, or Osgood–Schlatter disease may result in abnormal morphology of the tibial tuberosity. Additionally, this line does not change after tibial tuberosity distalization. In these cases, different measurement techniques may be required.

#### Modified Insall–Salvati

**Fig. 13:**
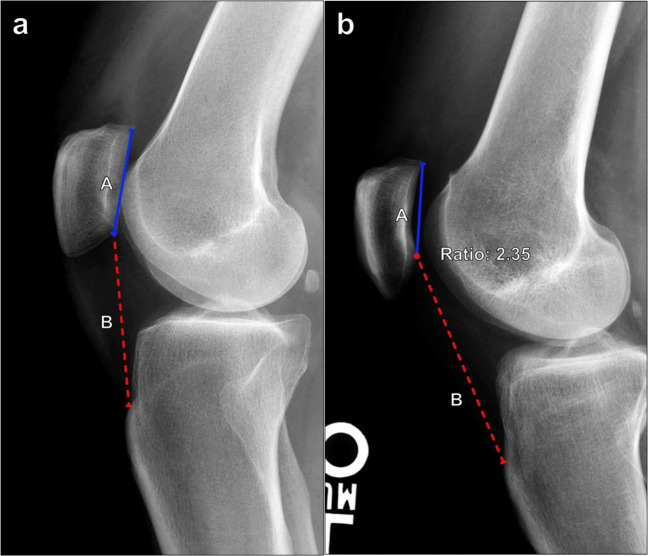
Measurement of the modified Insall–Salvati index on lateral knee radiograph. **a** The ratio is calculated as the distance from the inferior margin of the patellar articular surface to the patellar tendon insertion on the tibial tuberosity (B, red line) divided by the length of the patellar articular surface (A, blue line). A modified Insall–Salvati index should measure ≤ 1.2, and a ratio > 2 indicates patella alta. **b** Patella alta in the same patient as in Fig. 12b. The modified Insall–Salvati index measures 2.3, which is increased

In the case of abnormal patellar morphology, the modified Insall–Salvati ratio, also known as the Grelsamer–Meadows index, may be used (Fig. 13). Measured on lateral knee radiographs, the ratio compares the distance from the inferior margin of the patellar articular surface to the patellar tendon insertion on the tibial tuberosity and the length of the patellar articular surface. Normally, this measures 1.2–2.0, and a ratio of > 2.0 reflects patella alta [24]. In practice, the original and modified Insall–Salvati methods are largely equivalent.

#### Caton–Deschamps index

**Fig. 14:**
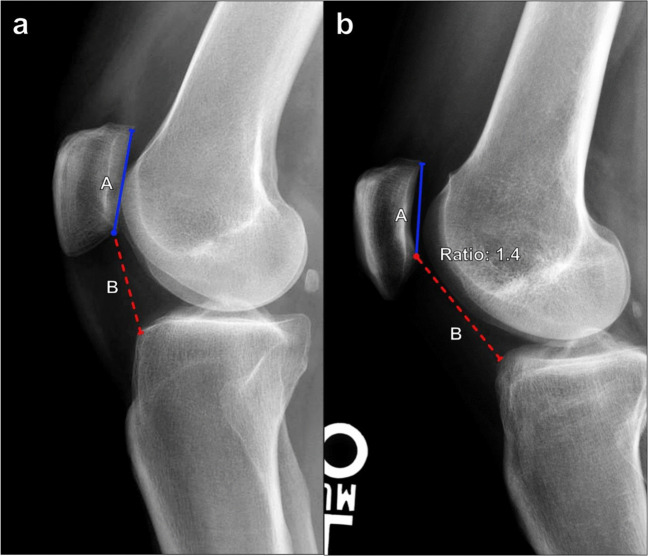
Measurement of the Caton–Deschamps index on lateral knee radiograph with the knee in 30° of flexion. **a** The ratio is calculated as the distance from the inferior margin of the patellar articular surface to the anterior margin of the tibial articular surface (B, red line) divided by the length of the patellar articular surface (A, blue line). A Caton–Deschamps index of > 1.3 indicates patella alta, while a value < 0.6 reflects patella baja. **b** Patella alta in the same patient as in Fig. 12b. The Caton–Deschamps index measures 1.4, which is increased

This is thought to be the most accurate diagnostic method for evaluating patellar height because it relies on readily identifiable anatomic landmarks, is not affected by the degree of knee flexion or radiograph quality, and can still be used after distalization of the tibial tubercle [25]. On a lateral radiograph of the knee, the patellar height is measured between the inferior margin of the patellar articular surface and the anterior margin of the tibial articular surface, while the patellar length is measured from the superior to the inferior margin of the patellar articular surface (Fig. 14). The index is the patellar height divided by the patellar articular surface length and normally equals 1. A Caton–Deschamps index of > 1.3 indicates patella alta, while a value of < 0.6 indicates patella baja [26]. This index can be measured on a sagittal MRI or CT reformatted imaging of the knee through the central patellofemoral joint; if the radiologist knows the referring clinician needs to assess patellar height prior to image acquisition, the study should be performed with the knee flexed to about 30° to standardize the measurement technique.

#### Less commonly used methods to measure patellar height

##### Blackburne–Peel

**Fig. 15:**
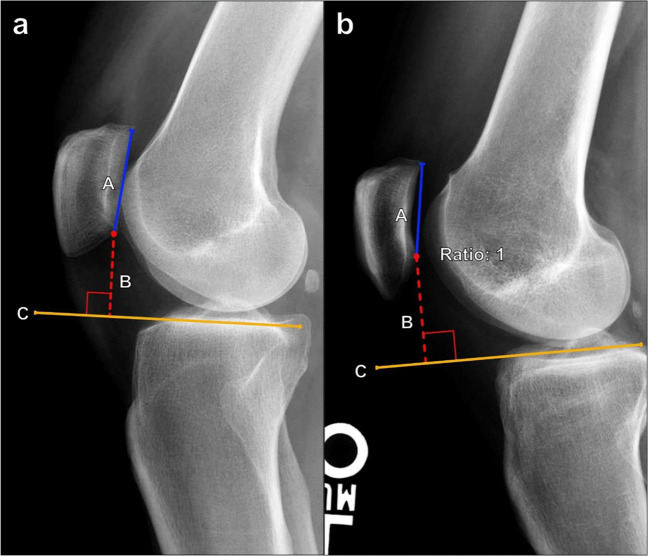
Measurement of the Blackburne–Peel ratio on lateral knee radiograph with the knee in 30° of flexion. **a** First, the length of the patellar articular surface is measured (A). Then, a horizontal line is drawn along the tibial plateau articular surface (C). Finally, a line (B) is made from the inferior border of the patellar articular surface extending and perpendicular to line C. The ratio is calculated as B/A. A normal value is 0.5–1.0, while values > 1.0 suggest patella alta. Limitations of this method include variations in inclination of the tibial plateau and projection angles which produce measurement inaccuracies. This measurement is not commonly used clinically since it shows greater interobserver variability than the other measurements. **b** Borderline patella alta in the same patient as in Fig. 12b. The Blackburne–Peel index measures 1.0, which is top normal. However, the other three indices clearly suggest patella alta in this patient

Described in detail in Fig. 15 [27, 28], the Blackburne–Peel ratio is rarely utilized in determining patellar height. Like the previously described methods, it is also assessed on lateral knee radiographs with 30° of knee flexion, although it can also be adapted to sagittal CT reformatted images and MRI. The normal range is 0.5–1.0. This method offers higher interobserver reliability than the Insall–Salvati index, particularly in cases with tibial tuberosity abnormalities, such as traction enthesophytes, or following knee arthroplasty [29].

##### Patellotrochlear index

**Fig. 16:**
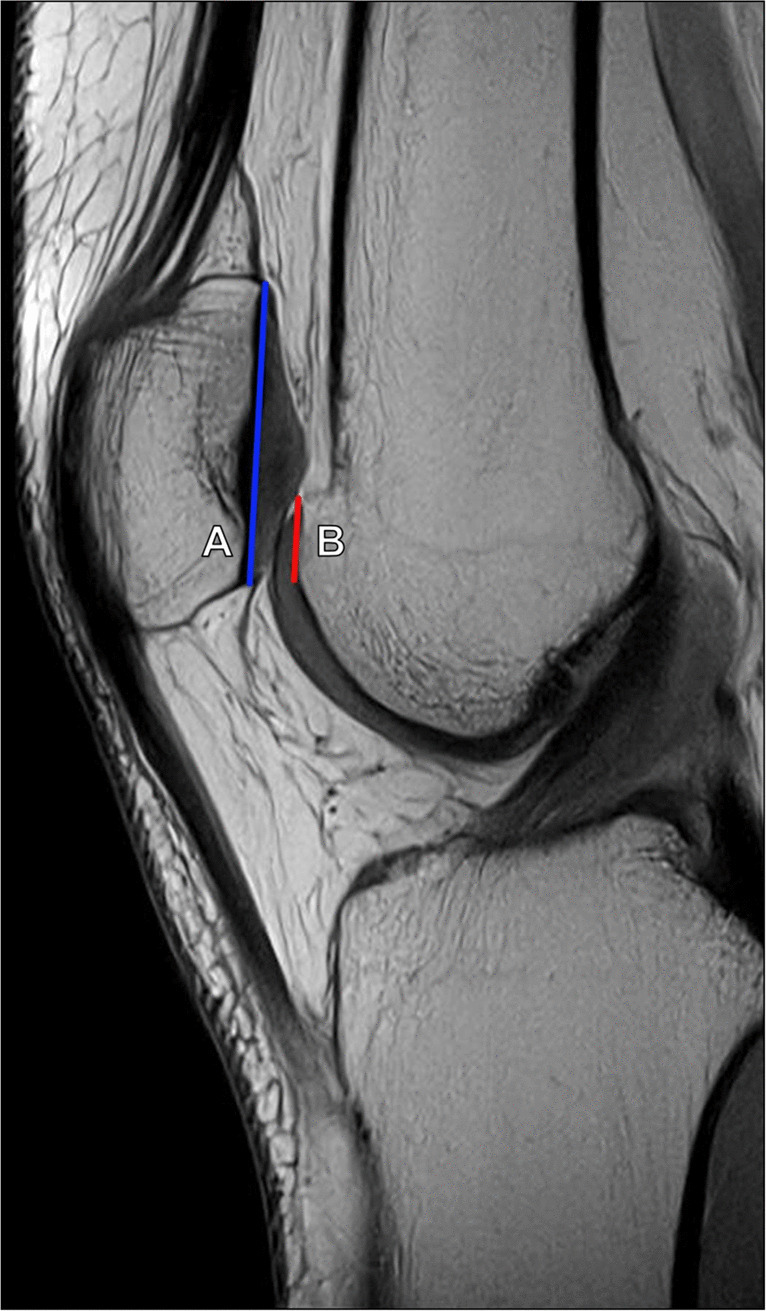
Measurement of the patellotrochlear index (PTI) on sagittal MR image. Measurement should be performed on the midline sagittal MR section through the patella with the thickest articular cartilage and maximal length of the patellar bone. The ratio is calculated as the length of the trochlear cartilage which overlaps the patellar cartilage (B, red line) divided by the total length of the patellar cartilage (A, blue line). The average ratio is 0.32 with ratios < 0.12–0.28 indicating patella alta. Patella baja was originally defined as a PTI > 0.50; however, newer studies suggest a cutoff of > 0.80

Unlike the above-described methods of patellar height measurement which utilize bony landmarks, the patellotrochlear index measures the actual articular congruence between the patella and distal femur. It is measured on sagittal MRI at the level of the maximal patellar cartilage thickness and calculated as the ratio of the trochlear cartilage overlap to the total length of the patellar cartilage. A ratio of < 0.12–0.28 indicates patella alta, while a ratio > 0.5–0.8 is compatible with patella baja. Proper sagittal slice selection is important to reduce interobserver variability (Fig. 16) [30, 31].

#### Anterior tibial subluxation

**Fig. 17:**
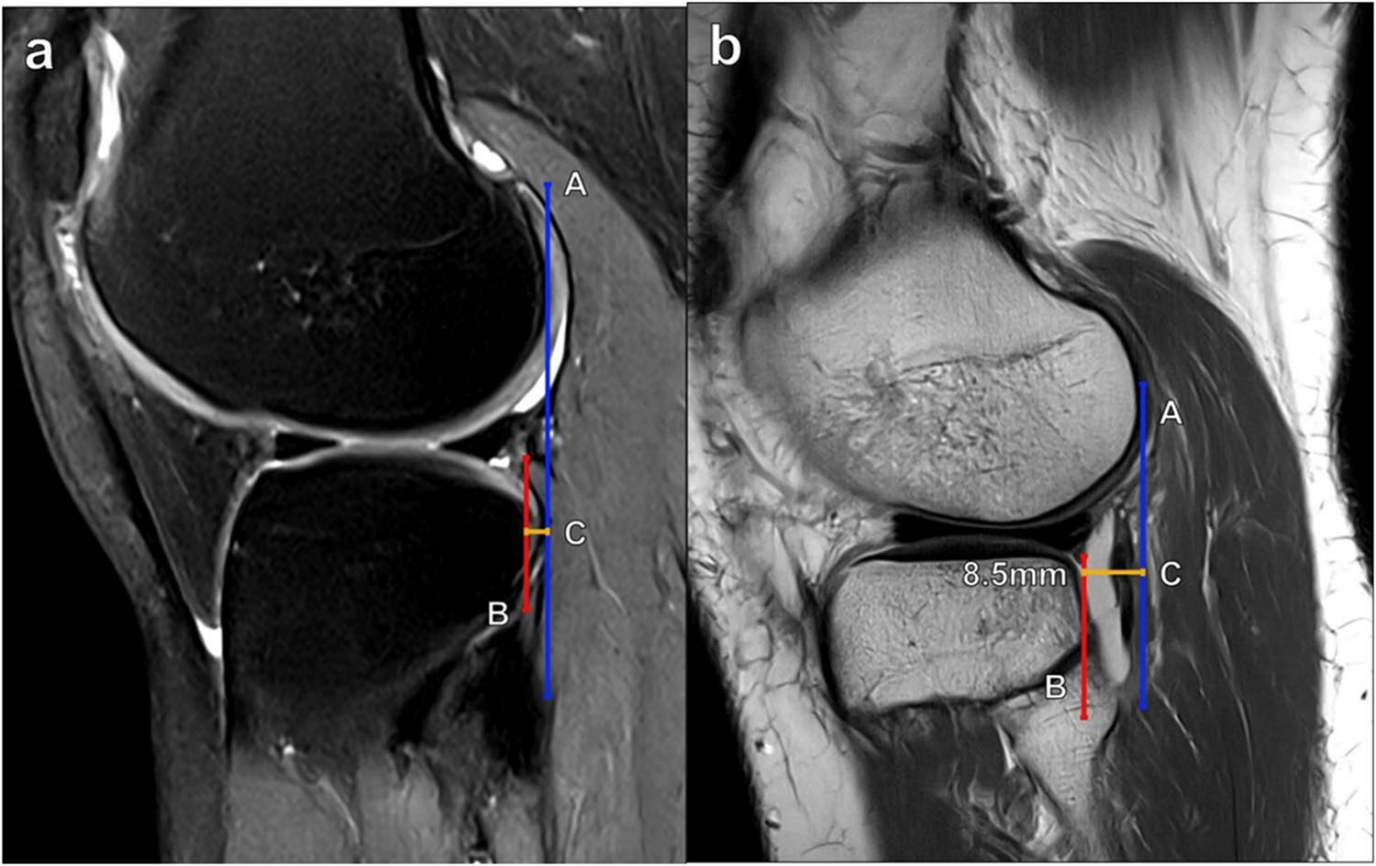
Measurement of anterior tibial subluxation on sagittal MR images. **a** Measurement should be performed on the sagittal MR section through the lateral compartment at the level of the mid-lateral femoral condyle where the diagnostic accuracy is the highest. A plumb line is drawn along the posterior lateral femoral condyle subchondral bone (A, blue line), and a second plumb line is drawn along the posterior lateral tibial plateau cortex (B, red line). The distance between the two lines (C, yellow line) is the degree of tibial subluxation. The value normally measures ≤ 5 mm, and any measurement > 7 mm is consistent with anterior tibial subluxation. **b** Sagittal proton-density weighted nonfat-suppressed image of the mid-lateral compartment in a 24-year-old male patient with a basketball injury demonstrates anterior tibial subluxation measuring 8–9 mm, consistent with anterior cruciate ligament insufficiency

Anterior knee instability, usually related to anterior cruciate ligament (ACL) insufficiency, is generally determined clinically rather than on imaging. However, in the setting of more pronounced instability, anterior tibial subluxation with respect to the distal femur can be seen on MRI, which is also known as the MRI anterior drawer sign. To measure the degree of anterior tibial subluxation, a sagittal MR image through the mid-lateral compartment of the knee is used, where the measurement has the highest diagnostic accuracy. Two vertical plumb lines are drawn, one from the posterior lateral femoral condyle subchondral bone plate and the other from the posterior cortex of the lateral tibial plateau (Fig. 17). The measurement has the highest diagnostic accuracy at the lateral femoral condylar level. Typically, the distance between the two lines measures ≤ 5 mm, with greater values associated with ACL tear or laxity [32, 33]; a study by Vahey et al. found anterior subluxation of > 7 mm was highly specific for detecting ACL tears and recommended this as the threshold to determine anterior tibial subluxation [33]. This measurement can also be helpful in serial knee MRI examinations, such as after ACL reconstruction, to determine whether there is progressive anterior instability and graft insufficiency [34].

#### Tibial tuberosity-trochlear groove distance

When the tibial tuberosity is more laterally positioned with respect to the trochlear groove, it may exaggerate lateral patellofemoral forces, increasing the risk for patellofemoral maltracking and accelerated patellofemoral cartilage degeneration. The tibial tuberosity-trochlear groove (TT-TG) distance measures the degree of tibial tuberosity lateral displacement relative to the trochlea and is an important measurement for preoperative planning in patellofemoral realignment; the larger the TT-TG distance, the greater the degree of correction required to reduce lateral forces on the patella. Factors that may contribute to an increased TT-TG distance include femoral anteversion and external tibial torsion [5].

**Fig. 18:**
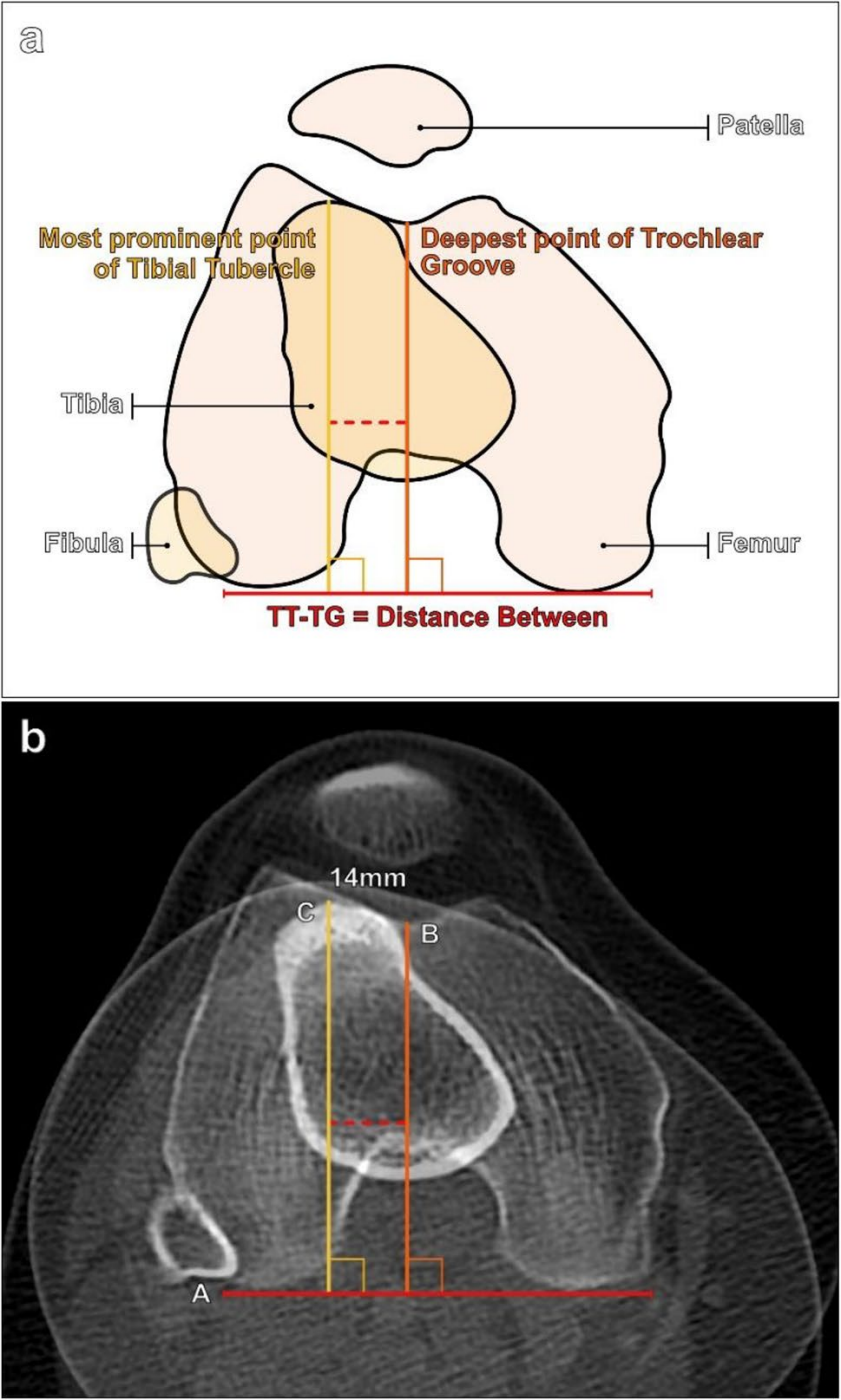
Measurement of the tibial tuberosity-trochlear groove (TT-TG) distance shown schematically and on clinical images. **a** Diagram depicting the method of assessing the TT-TG distance by superimposing an axial image of the knee through the femoral trochlea located approximately 3 cm above the femorotibial joint line and an axial image through the tibial tuberosity. First, a line connecting the posterior femoral condyles is drawn (solid red line). Then, a perpendicular line to the posterior bicondylar line is drawn on the femoral trochlear image through the deepest part of the trochlear sulcus (orange line). Next, a second line perpendicular to the bifemoral condylar line (yellow line) is drawn on the tibial tuberosity image to the apex of the tibial tuberosity, and the TT-TG distance is the distance between the orange and yellow lines (dashed red line). **b** On superimposed axial CT images of a 35-year-old male patient, A, solid red line, represents the posterior bicondylar line; B, orange line, is perpendicular to (A) and extends through the deepest part of the trochlear sulcus; and C, yellow line, is the perpendicular line extending through the tibial tuberosity. The TT-TG distance (dashed red line) measures 14 mm. The TT-TG distance normally measures < 15 mm. Values between 15 and 20 mm are considered borderline, while a TT-TG > 20 mm is abnormal

The TT-TG distance is measured on axial cross-sectional images of the knee (Fig. 18). First, a line is drawn tangent to the posterior femoral condyles. Subsequently, two lines are drawn perpendicular to the posterior condylar line: a line through the deepest part of the trochlear sulcus and a line through the most anterior portion of the tibial tuberosity. The distance between line B and line C is the TT-TG distance [5].

A normal TT-TG distance is < 15 mm, while a TT-TG > 20 mm is abnormal [5]. Values between 15 and 20 mm are considered borderline increased and should be interpreted in combination with additional imaging findings and clinical symptoms. Of note, CT is the gold standard for TT-TG assessment. Although the TT-TG distance can be measured on MRI, the values obtained when using MRI have been shown to underestimate the TT-TG distance by approximately 4 mm when compared to CT [35]. The difference may be due to a few factors, including slight knee flexion on MRI compared to full knee extension on CT, and the inclusion of soft tissue structures, such as articular cartilage and the patellar tendon insertion when making measurements on MRI.

### Arthroplasty

The objective of total knee arthroplasty (TKA) is to restore normal anatomy, motion, and stability of the knee. The anterior flange of a correctly sized femoral component should be parallel to and flush against the distal femoral anterior cortex. Oversized components can cause excessive soft tissue tension and decreased range of motion, while undersized components can result in notching of the anterior femoral cortex, which predisposes to periprosthetic supracondylar femoral fracture [36]. The size of the tibial component should qualitatively match the size of the native plateau; an oversized component with overhang may irritate adjacent soft tissues, and undersizing increases the risk of subsidence [37].

#### Radiographic and cross-sectional assessment

The minimum radiographic assessment includes properly positioned AP and true lateral radiographs of the knee with the X-ray beam centered at the joint line in order to avoid excessive rotation or joint flexion and avoid beam angle artifact, which can significantly distort angular measurements and obscure subtle signs of loosening or malalignment. Furthermore, baseline radiographs are important to detect early signs of prosthetic migration or wear that may only be visible on serial imaging studies.

**Fig. 19:**
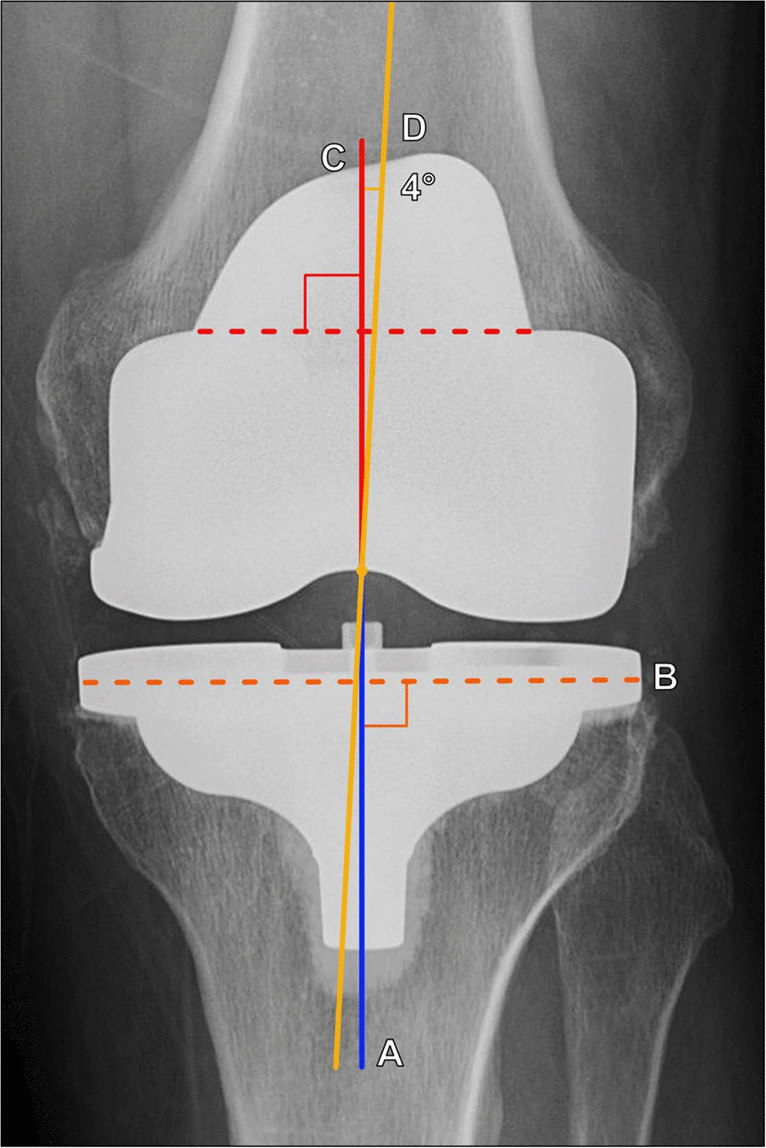
AP radiographic evaluation of a total knee arthroplasty. The tibial component articular surface (B, dashed orange line) is 90° relative to the tibial anatomic axis (A, blue line through the proximal tibia). The femoral component long axis (C, solid red line) is the perpendicular line to the femoral component articular surface (dashed red line). Relative to the femoral anatomic axis (D, yellow line through the distal femur), it should be 7° ± 3° valgus angulation, in a classic arthroplasty alignment. In this example, this angle measures 4°

When evaluating a classically aligned TKA on an AP radiograph (Fig. 19), the femoral component should be 7° ± 3° valgus to the femoral anatomic axis, and the tibial component should be 90° ± 3° to the tibial anatomic axis [36]. This allows for an overall 4°–7° valgus angulation of the knee using the anatomic axis of the femur and tibia. There has been recent increased interest in utilizing kinematic alignment techniques for TKA placement, in which the arthroplasty is placed with the goal of reestablishing the pre-arthritic alignment of the knee; in these cases, the radiographic alignment of the TKA may fall outside of these classic parameters [38].

**Fig. 20:**
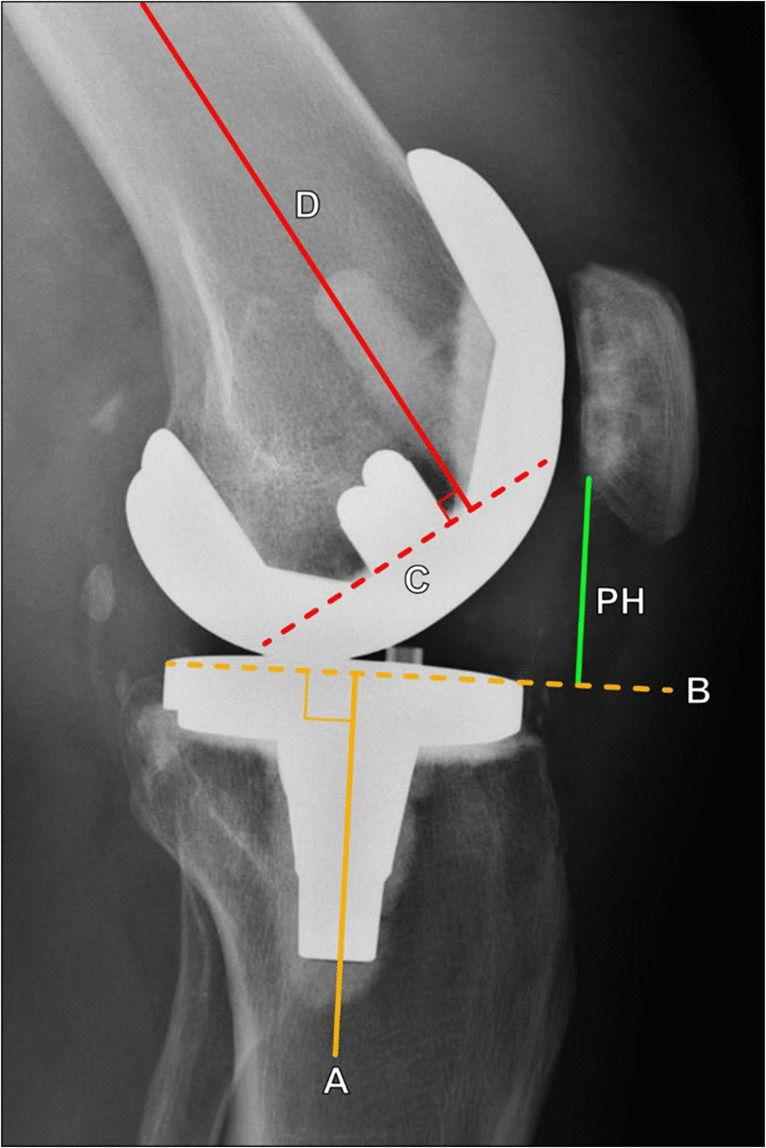
Lateral radiographic evaluation of a total knee arthroplasty. The tibial component articular surface (B) should be 90° relative to the tibial anatomic axis (A, solid yellow line) or slope posteriorly 3°–7°. The horizontal component of the femoral component (C, dashed red line) should be 90° relative to the femoral anatomic axis (D, solid red line). The patellar height (PH, green line) is measured perpendicular to a line tangent to the tibial articular surface (B, dashed yellow line) and is measured from this line to the inferior edge of the patellar component. The patellar height should measure 10–30 mm

On lateral knee radiographs (Fig. 20), the horizontal portion of the femoral component should be 90° relative to the anatomic axis of the femur [36]. The articular surface of the tibial component should be 90° relative to the tibial anatomic axis or slope posteriorly 3°–7°, and the post should be either central or posterior to the central tibial shaft [37]. The patellar height is measured along a line extending inferiorly from the inferior margin of the patellar component perpendicular to a line drawn along the tibial articular surface line and should be 10–30 mm [39].

**Fig. 21:**
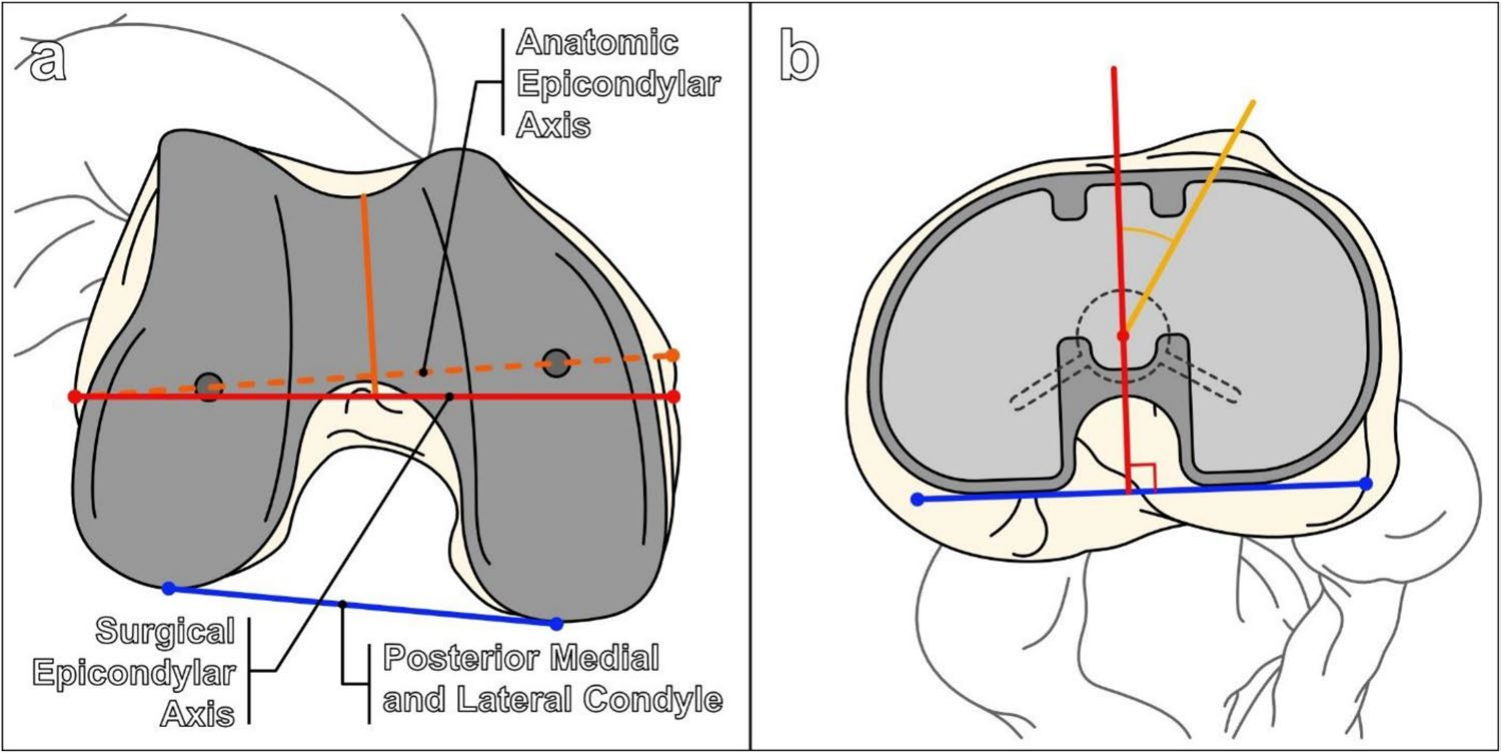
Diagrams illustrating the rotational axes of the distal femur and proximal tibia used in assessment of total knee arthroplasty. **a** Diagram of the femoral prosthetic component viewed from a bottom-up perspective. The distal femoral anatomic axis (dashed orange line) connects the apices of the medial and lateral femoral epicondyles, while the surgical epicondylar axis (red line) connects the medial femoral condylar sulcus with the lateral femoral epicondyle apex. The femoral surgical epicondylar axis is generally externally rotated compared to the anatomic axis. It is felt to represent the more physiologic axis for knee flexion and is more commonly used in determining placement of the femoral component. The anteroposterior femoral axis (solid orange line) is determined by a line connecting the deepest parts of the femoral trochlear sulcus and the intercondylar notch. The degree of femoral component rotation is made by comparing the position of the posterior condylar line (blue line) with the surgical epicondylar line, and the femoral component is usually slightly internally rotated. **b** Diagram of the tibial prosthetic component from a top-down perspective. The blue line represents a line connecting the posterior medial and lateral margins of the spacer. The red line is perpendicular to the blue line and extends through the center of the tibial post. The yellow line connects the center of the tibial tuberosity and the center of the tibial post, and the angle is taken between the red and yellow lines. The tibial component rotation is usually internally rotated with respect to the tibial tuberosity and obtained by making an angle between the yellow and red lines

**Fig. 22:**
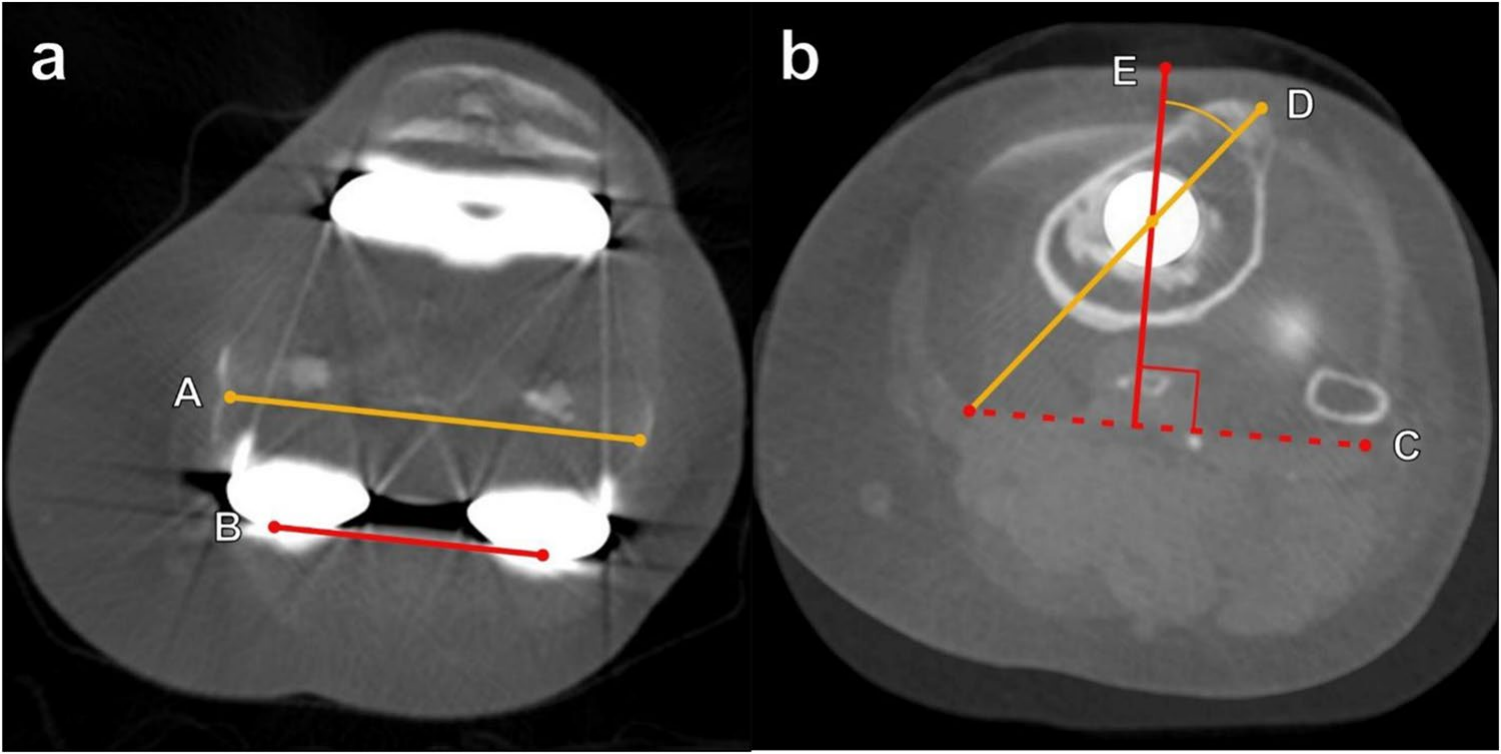
Evaluation of total knee arthroplasty on axial CT with metal artifact suppression technique. **a** Rotation of the femoral component is reported as the angle between a line drawn from the peak of the lateral epicondyle to the sulcus of the medial epicondyle (A, yellow line) and a line connecting the medial and lateral posterior prosthetic condylar surfaces (B, red line). The femoral component should be slightly internally rotated, and normal values are 0.3° ± 1.2° in women and 3.5° ± 1.2° in men. **b** Rotation of the tibial component is reported as the angle between a line (E, solid red line) perpendicular to the posterior tibial component at the level of the polyethylene liner (C, dashed red line) and a line from the center of the tibial component post to the center of the tibial tubercle (D, yellow line). The tibial component should be internally rotated approximately 18° ± 2.6° relative to the tibial tubercle. Figure (**b**) was obtained by superimposing axial images at the levels of the femorotibial spacer and the tibial tuberosity. Excessive internal rotation may predispose the patient to patellofemoral complications

Rotational alignment of the components is assessed on axial cross-sectional CT images (Figs. 21 and 22). The femoral component should be slightly internally rotated relative to the transcondylar axis. This is measured as the angle between a line drawn from the peak of the lateral epicondyle to the sulcus of the medial epicondyle (surgical femoral epicondylar axis) and a line connecting the posterior prosthetic medial and lateral condylar surfaces (Figs. 21a and 22a). Normal values are 0.3° ± 1.2° in women and 3.5° ± 1.2° in men [40]. The tibial component should be internally rotated approximately 18° ± 2.6° relative to the tibial tubercle. This is measured as the angle between a line bisecting the tibial tubercle through its center point and a line perpendicular to the posterior tibial component at the level of the polyethylene liner (Figs. 21b and 22b) [40].

**Fig. 23:**
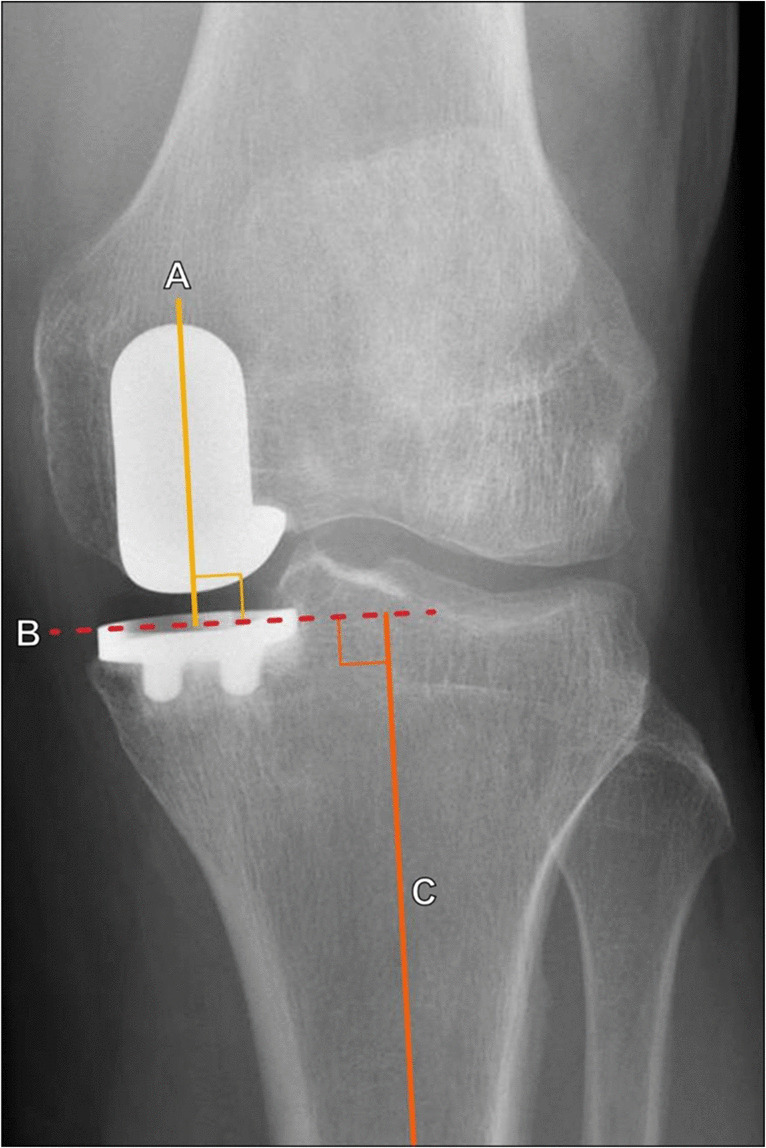
Radiographic evaluation of unicompartmental knee arthroplasty on AP radiograph of the knee. The long axis of the femoral component (A, yellow line) should be perpendicular to the tibial component articular surface (B, dashed red line), which should be perpendicular to the tibial anatomic axis (C, orange line). As a result, the angle between lines A and C should either be neutral (0°) or in slight valgus position

In unicompartmental arthroplasty, AP radiographs should demonstrate the tibial component articular surface perpendicular to the anatomic axis of the tibia and the femoral component perpendicular to the tibial component articular surface (Fig. 23) [41]. The valgus alignment should be neutral or slightly under-corrected [42]. On lateral radiographs, the tibial component should match the slope of the native tibia [41].

#### Postoperative complications

Many common postoperative complications following TKA, including early infection, deep venous thrombosis, arthrofibrosis, neurovascular injury, and persistent pain, are not measurable on radiologic studies. However, imaging can provide insight into septic or aseptic device loosening, soft tissue insufficiency, or implant wearing. Regardless of alignment technique, the medial and lateral polyethylene joint spaces should be equivalent. Gapping indicates ligamentous or capsular instability, while narrowing indicates wear of the spacer. Post-arthroplasty imaging should also be closely evaluated for signs of aseptic loosening, which is the most common long-term complication. Hardware loosening manifests as > 2 mm of periprosthetic lucency, and any new or increasing periprosthetic lucency should be closely scrutinized and followed. Loosening of the tibial component of a TKA is more common than loosening of the femoral component; over time, this can result in tilting of the tibial component with increasing varus angulation of the knee, as well as subsidence of the tibial component into the medial tibial plateau [37]. External rotation of the components is generally well tolerated, but internal rotation > 5° leads to patellofemoral complications [43].

## Conclusion

As demonstrated in this review article, the ability to accurately and reproducibly make and interpret measurements on diagnostic imaging of the knee is an important skill for the interpreting radiologist as well as the practicing orthopedic surgeon. Knowledge of quantitative measurements that can be made on diagnostic imaging of the knee often guides diagnosis, treatment options, and surgical management of many disorders. Although the interpreting radiologist should not rely exclusively on measurements to make diagnoses, such assessments can increase diagnostic confidence and improve communication of relevant conditions between radiologists and treating surgeons.

## Data Availability

Not applicable.
